# Pathway to Access UTI Management (PATH-UTI): evaluating pharmacist prescribing and management practices for uncomplicated urinary tract infections in community pharmacy in New South Wales and the Australian Capital Territory, Australia

**DOI:** 10.1007/s11096-026-02226-9

**Published:** 2026-09-23

**Authors:** Mitchell Budden, Francisco Martinez-Mardones, Shalom I. Benrimoj, Anna Campain, David Peiris, Sarah Dineen-Griffin

**Affiliations:** 1 Pharmacist Prescribing and Advanced Practice Research and Education Centre for Excellence, University of Newcastle, Callaghan, Australia; 2 Graduate School of Health, Faculty of Health, University of Technology Sydney, Sydney, Australia; 3 Pharmaceutical Care Research Group, University of Granada, Granada, Spain; 4 The George Institute for Global Health, UNSW Sydney, Sydney, Australia; 5 College of Health, Medicine and Wellbeing, School of Biomedical Sciences and Pharmacy, The University of Newcastle, University Drive, Callaghan, NSW 2308 Australia

**Keywords:** Community pharmacist, Community pharmacy service, Evidence-based pharmacy practice, Non-medical prescribing, Pharmacist, Urinary tract infection

## Abstract

**Introduction:**

Pharmacist prescribing has been introduced internationally to improve timely access to treatment in primary care. For uncomplicated urinary tract infections, pharmacist-led services report favourable outcomes, however, the evidence base remains limited by small sample sizes and heterogenous study designs. Large-scale evaluations are needed to better understand pharmacist management practices and prescribing outcomes.

**Aim:**

To explore pharmacist management practices for uncomplicated urinary tract infections in community pharmacy settings, including the effectiveness and safety of pharmacist prescribing and variation in clinical outcomes by antibiotic type.

**Method:**

Data from the PATH-UTI cohort study were analysed across community pharmacies in New South Wales and the Australian Capital Territory. The trial included an initial two-month feasibility pilot followed by a 10-month main study period (July 2023–May 2024). Women aged 18–65 years presenting with symptoms consistent with an uncomplicated urinary tract infection were assessed and managed by trained pharmacists using a structured clinical management protocol aligned with national guidelines. Consultation data were routinely recorded during the pharmacy visit, and patient-reported outcomes were collected by follow-up survey 7 days later. The primary outcome was self-reported complete symptom resolution at 7-day follow-up. Secondary outcomes included antibiotic prescribing rates, patient reported adherence, referrals, and patient reported adverse events. Differences in symptom resolution by antibiotic type were assessed using a chi-square test of independence.

**Results:**

A total of 18,143 consultations were recorded, with 14,680 follow-up surveys (81.0%) completed. Antibiotics were supplied in 92.6% of consultations, most commonly trimethoprim (95.5%). Complete symptom resolution at 7 days was reported by 79.4% of patients. A further 17.9% reported partial symptom improvement. There was no statistically significant difference in effectiveness across the three antibiotics (χ^2^(2) = 3.88, *p* = 0.14). Referrals to a general practitioner or emergency department occurred in 6.9% of cases. Self-reported adverse events were documented in 5.0% of patients, predominantly mild gastrointestinal side effects.

**Conclusion:**

Pharmacist prescribing for uncomplicated urinary tract infections achieved symptom resolution rates comparable to previous pharmacy- and physician-led studies. Low rates of adverse events and complications, and strong adherence to the clinical management protocol were also observed. These results suggest that structured, protocol-based pharmacist prescribing for uncomplicated urinary tract infections can support safe and effective management in primary care, with the potential to improve access to treatment and help manage demand on general practice.

*Trial Registration*: Retrospectively registered: ACTRN 12623000882628.

**Supplementary Information:**

The online version contains supplementary material available at https://doi.org/10.1007/s11096-026-02226-9.

## Impact statements

- Pharmacist management of uncomplicated urinary tract infections achieved high rates of symptom resolution in real-world practice, supporting pharmacist prescribing as an effective model of care.
- Pharmacists demonstrated high adherence to clinical protocols, including appropriate antibiotic selection and use of referral pathways, supporting the safety and consistency of the prescribing model.
- Symptom resolution and adverse events were examined across antibiotic treatments, with no statistically significant difference in effectiveness identified between antibiotics.
- This study provided scalable evidence to support the wider adoption of pharmacist prescribing models in primary care, with potential to improve timely access to treatment and help manage demand on general practice.

## Introduction

The evolving scope of pharmacy practice has prompted significant discourse regarding the role of pharmacists and its implications for patient care [1–4]. A key development has been the growing international recognition by governments of pharmacist prescribing and expanded scope of practice. Historically, the authority to prescribe medications has been largely restricted to a select group of healthcare professionals, primarily general practitioners (GPs) and physicians, with limited prescribing rights extended to some other professions [5–8]. However, ensuring timely access to safe and effective care in primary care has emerged as a critical priority for health systems [6]. Many jurisdictions, including Australia, Canada, New Zealand (NZ), the United Kingdom (UK), and the United States (US), have introduced legislative changes enabling pharmacists to prescribe within a defined scope of practice [9–15]. Various models for pharmacist prescribing have emerged [8, 12, 16–18].

A systematic review examining the impact of pharmacist prescribing on access to medicines concluded that it enabled timely and convenient access to treatment and received positive feedback from consumers [19, 20]. Stewart’s qualitative study found similarly positive sentiments regarding convenience and after-hours access when medical practices were closed or when patients could not obtain a physician appointment [20]. Similarly, a Canadian study identified several benefits of pharmacist prescribing, including enhanced access to healthcare services, increased efficiency of the health system, and improved patient outcomes [21].

The evidence base supporting pharmacist prescribing continues to evolve, with much of the current literature focused on establishing the accessibility, safety, effectiveness and acceptability of these services [8, 12, 16–18]. Studies examining pharmacist prescribing for uncomplicated urinary tract infections (uUTIs) have asserted that the practice can be both safe and effective, reporting consistently high symptom resolution rates (84–89%), low rates of retreatment and adverse events, and high levels of patient satisfaction [22–24]. However, many studies are limited by small sample sizes, variable study designs, and differences in clinical management protocols, which limit generalisability [22, 24–27].

While further robust evaluation may be warranted, pharmacist-led management and treatment of uUTIs has already been incorporated into broader healthcare reforms in many jurisdictions and is now routine practice in Australia [27–31], Canada [32–36], France [37], Ireland [38], NZ [39], Switzerland [40], the UK [41–48] and the US [49, 50]. The legal mechanisms through which pharmacists provide treatment, including prescribing or medicine supply under other authorised mechanisms, vary between and within countries [51]. Differences in patient eligibility criteria, diagnostic methods, and treatment protocols have been reported, with a recent review highlighting substantial variation in the content and quality of clinical management protocols (CMPs) for pharmacist prescribing of uUTIs [26, 27].

In Australia, legislative authority for pharmacist prescribing is determined at the state and territory level, with jurisdictions progressively implementing models that enable appropriately trained pharmacists to prescribe for specified conditions and services [2, 30]. Recognising the potential for community pharmacists to play an expanded role in primary care, New South Wales (NSW) and Australian Capital Territory (ACT) have introduced initiatives aimed at improving access to healthcare in Australia [52]. These include funding pharmacist prescribing trials for the management of uUTI, resupply of hormonal contraception and management of dermatological conditions [26, 52–55]. The Pathway to Access UTI Management (PATH-UTI) trial was the first of these initiatives, enabling appropriately trained pharmacists to assess and prescribe treatment for eligible patients with uUTI under the relevant jurisdictional arrangement [54].

### Aim

To explore pharmacist management practices for uncomplicated urinary tract infections in community pharmacy settings, including the effectiveness and safety of pharmacist prescribing and variation in clinical outcomes by antibiotic type.

## Method

Data from the PATH-UTI cohort study were analysed to explore pharmacist management practices, including antibiotic selection, effectiveness and safety of pharmacist prescribing for uUTI in community pharmacies across NSW and in selected pharmacies in the ACT, Australia. The trial commenced with an initial two-month feasibility pilot study, followed by a 10-month main study period (July 2023 to May 2024).

### Eligibility, recruitment and consent

All community pharmacies in NSW by March 2023 (n = 2012) were invited to participate through an open expression of interest (EOI) process [54]. The NSW Ministry of Health developed an EOI form, which could be submitted via email to the NSW Pharmacy Trial mailbox [54]. EOI forms were then assessed by the research team to determine whether they met the eligibility criteria for participation in the project (Table 1; Supplementary material 1). A formal recruitment letter, participant information sheet, and consent forms were emailed to eligible pharmacies (Supplementary material 2). Pharmacy owner consent was obtained electronically via DocuSign [56]. Consenting pharmacies were required to nominate pharmacists to participate in the study. Eligible pharmacists were required to provide voluntary consent electronically via DocuSign [56]. A total of 1528 pharmacies expressed initial interest to participate in the trial, for whom 1320 consented to participation.

**Table 1:** Summary of pharmacy, pharmacist and patient eligibility criteria

Participant	Inclusion Criteria	Exclusion Criteria
Community pharmacies	Have a private consultation room or area that met the NSW Health Authority requirements, including: Not used as a dispensary, storeroom, staff room or retail space Fully enclosed with adequate privacy (curtains or dividers in unsuitable areas were not acceptable) Equipped with adequate lighting and maintained a comfortable ambient temperature Include hand sanitisation facilities and ready access to handwashing Sufficient floor space to accommodate the patient, a support person (if required), and the pharmacist Have access to MedAdvisor for clinical record keeping Have at least one eligible pharmacist who provided voluntary consent and was available to deliver the service during all pharmacy opening hours	Did not have a private consultation area or room that met the minimum requirements outlined by the NSW Health Authority Did not have access to the MedAdvisor PlusOne software Were unable to provide the service during all opening hours of the pharmacy
Pharmacists	Hold general registration with the Australian Health Practitioner Regulation Agency (Ahpra) Be employed or engaged at a participating pharmacy in NSW or ACT Have completed one of the following accredited training programs: Australasian College of Pharmacy (ACP), Uncomplicated Cystitis Accreditation number: A2303PGA1 [60]; **OR** Pharmaceutical Society of Australia (PSA)—Managing uncomplicated cystitis (UTI)—ACT, NSW, Qld, SA, Tas, WA & NT Accreditation Code: CONL22022 [61]; Complete additional training modules approved by the Chief Health Officer for the trial	Pharmacists were excluded if they held provisional registration (e.g., intern pharmacists) or had any conditions placed on their registration
Patients	Eligible patients were females aged 18–65 years who presented to a participating community pharmacy	Patients were excluded if they had undergone gender reassignment surgery or did not provide informed consent

For full details regarding the legislative instrument underpinning the service, see Supplementary material 1

ACT Health, the Government directorate responsible for public health policy, planning and health system oversight, released an open call for EOIs and stratified submissions by geographic region [57]. Initially, five pharmacies- one per region- were randomly selected by the project statistician [54]. An additional ten pharmacies were later added to the trial and were randomly selected, irrespective of geographic region.

The location of participating pharmacies was classified using the Modified Monash Model (MMM), which categorises areas by remoteness and population size on a scale from MM1 (metropolitan) to MM7 (very remote) [58].

Patient information materials designed by NSW Health were used to inform patients about the service [54]. Patients presenting with symptoms suggestive of a uUTI were asked to review the Participant Information Statement and consent information using their own electronic device, usually a mobile phone. A pharmacy-specific QR code directed patients to a secure electronic consent platform (GDS) hosted by The George Institute (TGI) for Global Health [59]. Patients who provided electronic informed consent were then able to proceed with the pharmacist consultation. A copy of the completed consent form was provided to the patient by email. Eligible patients were females aged 18–65 years who presented to a participating community pharmacy with symptoms suggestive of a uUTI.

### Intervention

Following consent and prior to service delivery, pharmacists were required to complete a clinical training course (either through the Australasian College of Pharmacy (ACP) [60] or the Pharmaceutical Society of Australia (PSA) [61]). The training equipped pharmacists with the knowledge required to distinguish between types of genitourinary infections, understand the underlying pathogenesis and common causative organisms, recognise the key clinical features of uUTIs, and select appropriate antibiotic therapy in accordance with the Australian Therapeutic Guidelines (eTG) [62, 63]. In addition, they were also required to complete study specific training modules designed to support the consultation process, patient consent and recruitment, timely referral, and quality data collection. Follow up training and ongoing support were provided by Practice Change Facilitators (PCFs), individuals with subject matter expertise who assist, mentor, coach and support implementation by working with community pharmacies and pharmacists [54, 64, 65]. As part of the implementation strategy, PCFs provided support through face-to-face visits and digital communications, responded to implementation queries, supported service delivery, and collected implementation-related data [54].

Pharmacist consultations followed a structured format using a purpose-designed MedAdvisor PlusOne software and co-designed CMP aligned with the eTG (Supplementary material 3) [54, 62, 63]. Consultations were anticipated to take approximately 10 min. Participating pharmacies received a payment of $25 per consultation as compensation for their participation in the study. This payment was provided for research participation and was not a fee for the consultation itself. Patients did not receive any payment for participating in the study and remained responsible for the cost of any medicines or other products supplied [54].

The diagnosis of uUTI was made where patients presented with at least two urinary symptoms suggestive of uUTI (i.e., dysuria, urinary frequency, urinary urgency, or suprapubic pain or discomfort) in the absence of vaginal discharge and/or itchiness. This approach is supported by evidence indicating that women who meet these diagnostic criteria have approximately 90% probability of having an acute uUTI [26, 62, 63, 66].

Where diagnostic and eligibility criteria were met, empirical treatment could be supplied in accordance with the CMP. Pharmacists could provide one of three antibiotics included in the study: trimethoprim as first-line therapy, administered once daily for 3 days; nitrofurantoin as second-line therapy, administered four times daily for 5 days; or cefalexin as third-line therapy, administered twice daily for 5 days [62, 63]. Conservative management, including symptomatic relief, self-care advice, and safety-netting recommendations, was offered either as standalone therapy or alongside antibiotics where clinically appropriate (Supplementary 3). Patients presenting with symptoms suggestive of an alternative diagnosis, such as vaginal thrush, were provided standard over the counter (OTC) treatment in line with usual care [26].

Patients presenting with high-risk conditions, including systemic symptoms (e.g., flank pain, vomiting, and fever) or a history of recurrent UTIs (defined as two or more within six months, or three or more within 12 months), were referred to a general practitioner (GP) or emergency department (ED). Patients with moderate-risk conditions and symptoms, such as risk factors for possible sexually transmitted infection (STI) or use of other medications that increase UTI risk, were eligible for treatment and/or referral to a GP depending on their clinical presentation (Supplementary material 3).

Seven days after the consultation, patients received a follow-up survey via SMS or email (Supplementary material 4) [54]. The survey was purpose-designed for the PATH-UTI study to collect prespecified clinical and patient-reported outcomes, including symptom resolution, adverse events, and medication adherence [54]. The survey and associated data-collection process were implemented during the two-month feasibility phase preceding the main trial; however, formal psychometric validation was not undertaken [54]. The 7-day timeframe was selected to align with expected symptom resolution for uUTIs and with follow-up periods used in prior studies evaluating pharmacist prescribing for uUTIs. Up to three reminder messages were issued at days nine, 11, and 13. Patients who did not complete the survey after these reminders were considered lost to follow-up [54].

### Study outcomes

The primary outcome of the trial was self-reported 7-day symptom resolution rate, assessed using the 7-day follow-up survey, in which patients indicated whether their symptoms were completely resolved, improved but not completely resolved, or not improved or worsened.

Secondary outcomes reported include: (a) the proportion of patients supplied antibiotics by pharmacists; (b) antibiotic adherence and completion of the prescribed course; (c) the proportion of first-line antibiotics supplied as a percentage of all antibiotic prescriptions; (d) the proportion of patients referred to another healthcare professional; and (e) the incidence and type of self-reported adverse events identified at the 7-day follow-up, which were categorised using the International Statistical Classification of Diseases (ICD-10) system and their rates were compared to those reported in national and international databases (Supplementary material 5).

Additional outcomes collected as part of the PATH-UTI trial, including patient-reported experience measures, economic evaluations, and antimicrobial resistance (AMR) outcomes, are not reported in this paper and are presented elsewhere [54].

### Statistical analysis

Descriptive statistics summarised pharmacy and patient characteristics, symptom presentation, risk categorisation, and management outcomes, including treatment approach, antibiotic use, adherence, and referral reasons.

A comparison across the three antibiotic medications was performed using a chi-square test of independence to evaluate whether treatment success differed between antibiotics. Each medication was coded as a categorical variable with two outcomes: completely resolved versus not completely resolved. A 3×2 contingency table was constructed, and the chi-square statistic was calculated using observed and expected frequencies. Assumptions for the chi-square test were met, as all expected cell counts exceeded conventional thresholds.

Multivariable analyses were not undertaken because the primary objective was descriptive evaluation of real-world service outcomes rather than casual estimation, and several clinically relevant covariates, such as symptom severity and prior antibiotic exposure, were not consistently available.

### Data collection and management

Data were collected from two primary sources. Community pharmacy data were entered by pharmacists into a purpose-designed MedAdvisor PlusOne software during the encounter. The data were securely stored on a central server hosted by MedAdvisor [54, 67]. Participant self-reported data were collected via the 7-day follow-up survey hosted on GDS case report forms by TGI [54, 59].

### Ethics approval

The PATH-UTI trial was approved by the University of Newcastle’s Human Research Ethics Committee (HREC) on 27 June 2023 (H-2023-0119). The trial was registered on the Australian-New Zealand Clinical Trials Registry (ANZCTR) under registration number ACTRN12623000882628 [68].

## Results

A total of 18,143 consultations were delivered, with 95.7% (n =17,356) conducted in NSW and 4.3% (n = 787) in the ACT. Follow-up patient data were obtained for 81.0% of consultations. The mean age of patients was 41.7 years (SD ± 13.8). Most consultations occurred in metropolitan and regional cities (73.9%) (Table 2).

**Table 2:** Patient characteristics (n = 18,143)

Characteristic	Patients prescribed an ABX		Patients not prescribed an ABX		Total	
	Mean	SD	Mean	SD	Mean	SD
Age, mean ± SD	42.6	±13.7	42.1	± 14.7	42.5	± 13.8
**Remoteness category (Modified Monash (MM))**^**a**^	**n = 16,806**	**%**	**n = 1337**	**%**	**n = 18,143**	**%**
Metropolitan, Regional (MM 1–2)	12,452	74.1	948	70.9	13,400	73.9
Large to Small Rural Towns (MM 3–5)	4351	25.9	389	29.1	4740	26.1
Uncomplicated UTI symptoms^b^						
Dysuria	12,103	72.0	880	65.8	12,938	71.6
Suprapubic pain or discomfort	6035	35.9	569	42.5	6604	36.4
Urinary urgency	12,152	72.3	840	62.8	12,992	71.6
Urinary frequency	15,250	90.7	1072	80.1	16,322	90.0
Number of UTI symptoms at presentation						
0 to 1 symptom	20	0.1	151	11.3	171	1.0
2 symptoms	7260	43.2	486	36.3	7746	42.7
3 symptoms	7104	42.3	451	33.7	7555	41.6
4 symptoms	2422	14.4	249	18.6	2671	14.7
Additional symptoms (suggestive of alternative diagnosis/complicated UTI)^b^						
Systemic symptoms^c^	0	0.0	432	2.4	432	2.4
Vaginal symptoms, such as itch or discharge^d^	44	0.3	237	17.7	281	1.5
Risk category^e^						
High-risk category (Red Flags)^e^	29	0.2	682	51.0	711	3.9
Moderate-risk category (Amber Flags)^e^	392	2.3	95	7.1	487	2.7

^a^Fewer than five patients were treated in Remote and Very Remote regions (MM 6–7)

^b^Multiple responses allowed

^c^Systemic symptoms were classified as high-risk in accordance with the CMP

^d^Vaginal symptoms were classified as moderate-risk in accordance with the CMP. Systemic symptoms were classified as high-risk in accordance with the CMP

^e^Total number of consultations with high-risk (n = 1143) and moderate-risk (n = 768) categories, excluding systemic and vaginal symptoms, which are reported separately above

The most frequently reported symptoms were urinary frequency (90.0%), urinary urgency (71.6%), and dysuria (71.6%). Most consultations reported two (42.7%) or three (41.6%) symptoms consistent with a UTI. A total of 1143 consultations (6.3%) presented with symptoms or conditions considered high risk (red flags) (Table 2; Supplementary material 3).

### Management outcomes

Following pharmacist assessment, 92.6% of consultations resulted in prescribing of empirical antibiotic therapy. Trimethoprim was the most prescribed antibiotic (95.5%), followed by cefalexin (2.7%) and nitrofurantoin (1.8%) (Table 3). Overall, 6.9% of patients (n = 1260) were referred to a GP or ED (Table 3).

**Table 3:** Management outcomes following pharmacist consultation: Treatment approach, antibiotic use, patient reported adherence and referral reasons (n = 18,143)

Outcome of consultation with pharmacist	n = 18,143	%
Empirical antibiotic therapy provided ± conservative management	16,669	91.8
Empirical antibiotic therapy and referral to GP ± conservative management	137	0.8
Conservative management only, i.e., OTC and/or non-pharmacological advice, ± no treatment provided	214	1.2
Referred to GP ± conservative management	1123	6.2
**Antibiotics provided by the pharmacist**	**n = 16,806**	**%**
Trimethoprim	16,044	95.5
Cefalexin	461	2.7
Nitrofurantoin	301	1.8
**Patient-reported adherence**	**n = 14,680 **^**a**^	**%**
Taken as prescribed	13,024	88.7
Trimethoprim taken for 3 days as prescribed ^b^	12,458	94.0
Cefalexin taken for 5 days as prescribed ^c^	336	91.3
Nitrofurantoin taken for 5 days as prescribed ^d^	230	89.8
Did not take	1437	9.8
**Reasons for referral**	**n = 1260**	**%**
Met referral-only criteria from treatment protocol ^e^	1062	84.3
Referral as part of the pharmacist treatment ^e^	185	14.7
Met referral criteria as part of the screening questions	13	1.0

^a^Of the 14,680 survey respondents, 219 reported taking an antibiotic as prescribed despite no antibiotic being prescribed through the pharmacist-led service. Of these, 122 subsequently attended another healthcare professional and reported receiving antibiotic treatment, while 97 could not be reconciled with the available data. These 219 responses were excluded from the antibiotic adherence analysis

^b^Total number of survey respondents prescribed trimethoprim (n = 13,260)

^c^Prescribed cefalexin (n = 368)

^d^Prescribed nitrofurantoin (n = 256)

^e^Referral criteria for moderate- and high-risk categories were defined according to the CMP (Supplementary material 3)

### Patient-reported outcomes

A total of 14,680 (81.0%) follow-up surveys were completed, with 88.7% (13,024/14,680) reporting that they took their medication as prescribed (Table 3). When stratified by antibiotic type, adherence was highest among those prescribed trimethoprim, with 94.0% (12,458/13,260) completing the 3-day antibiotic course.

Self-reported complete symptom resolution at 7 days was achieved in 79.4% of patients who completed the follow-up survey (11,662/14,680). An additional 17.9% (2621/14,680) reported partial resolution of their symptoms (Table 4).

**Table 4:** Effectiveness outcomes: Symptom resolution outcomes by treatment approach: Antibiotic and non-pharmacological management (n = 14,680 survey responders)

Symptom Resolution	Trimethoprim		Cephalexin		Nitrofurantoin		Non-prescription treatment *	
	n	%	n	%	n	%	n	%
Completely resolved	10,638	80.2	302	82.1	217	84.8	505	63.4
Improved but not completely resolved	2292	17.3	63	17.1	34	13.3	232	29.1
Not improved or worsened	330	2.5	3	0.8	5	2.0	59	7.4
Total	13,260	100	368	100	256	100	796	100

*Treatment provided could have been conservative (non-prescription medications such as urinary alkalinising agents or ibuprofen), a referral to another healthcare provider or no treatment provided at all

A total of 94.6% of survey responders received antibiotics (13,884/14,680). Overall, 80.4% (11,157/13,884) reported complete symptom resolution. Success rates were 80.2% for Trimethoprim (10,638/13,260), 82.1% for Cephalexin (302/368), and 84.8% for Nitrofurantoin (217/256). The chi‑square test indicated no statistically significant difference in effectiveness across the three medications (χ^2^(2) = 3.88, *p* = 0.14).

Among survey responders (n = 14,680), self-reported adverse events were documented in 5.0% (n = 741) of consultations (Table 5). Most adverse events were mild and self-limiting, with gastrointestinal events being the most frequently reported (61.5%). A smaller proportion of participants (22.7%) reported genitourinary events, most commonly vaginal thrush or lack of symptom improvement following therapy. Antibiotic-specific adverse event rates are provided in Table 5. A comparative analysis of the adverse events reported in this study is provided in Supplementary material 5, with the incidences being comparable to those reported by different sources of the literature.

**Table 5:** Incidence of adverse events reported in PATH-UTI (n = 14,680 survey responders)

ICD-10 category	Trimethoprim n (%)	Nitrofurantoin n (%)	Cefalexin n (%)	Conservative Management (Nil antibiotic) n (%)	Total of adverse events n (%)	Most commonly reported adverse events^a,b^
Gastrointestinal	400 (54.0)	27 (3.6)	10 (1.3)	19 (2.6)	456 (61.5)	Abdominal pain
						Constipation
						Diarrhoea
						Nausea
						Vomiting
Genitourinary	150 (20.2)	4 (0.5)	7 (0.9)	7 (0.9)	168 (22.7)	Lack of efficacy
						Thrush (vaginal)
Neurological	46 (6.2)	1 (0.1)	1 (0.1)	5 (0.67)	53 (7.2)	Fatigue
						Headache
Dermatological	31 (4.2)	–	2 (0.3)	2 (0.3)	35 (4.7)	Night sweats
						Skin rash
						Sunburn
Immunological	18 (2.4)	–	–	–	18 (2.4)	Allergy
Other type^c^	4 (0.5)	1 (0.1)	1 (0.1)	1 (0.1)	7 (0.9)	Epistaxis; fluid retention; Joint pain: Leg cramps
Respiratory	4 (0.5)	–	–	–	4 (0.5)	Difficulty breathing
						Flu-like symptoms
						Fever
Total^d^	653 (4.9%)	33 (12.9%)	21 (5.7%)	34 (4.2%)	741 (100)^e^	
Percentage (%) of adverse events or complications reported in PATH-UTI (n = 14,680)					5.0	

^a^Detailed analysis for each adverse event (AE) is in Supplementary material 5

^b^Adverse events were reported as free-text responses and categorised using the ICD-10 classification systems

^c^Patients who indicated the presence of an AE but provided no details (e.g., “nil” or “none”) were excluded from analysis (n = 7)

^d^Proportion of adverse events by antibiotic type (%)

^e^Multiple adverse events could be reported within a single consultation; however, analyses were conducted at the consultation level, with each consultation counted once if any adverse event was reported

## Discussion

The PATH-UTI trial generated further evidence of the potential contribution of community pharmacy in managing healthcare demand among women presenting with uUTIs. Participation from 1320 of approximately 2012 community pharmacies in NSW (65.6%), of which 1028 (77.8%) recruited at least one patient, indicates that pharmacist-led prescribing services for uUTIs can be implemented across routine community pharmacy practice.

### Primary outcome: symptom resolution

Symptom resolution rates observed in this study are consistent with previous studies evaluating pharmacist prescribing for uUTIs, which have reported resolution rates between 75 and 91% [24, 27, 69–72]. Importantly, comparable outcomes have also been observed in physician-led care, where resolution rates range from 67 to 95% [73–82]. Chi-square analysis demonstrated no significant difference in effectiveness across the three antibiotics (χ^2^(2) = 3.88, *p* = 0.14), indicating that the observed variation in symptom resolution rates is likely attributable to random sampling variation rather than true differences in treatment effectiveness.

Interpretation of antibiotic effectiveness should also consider evolving AMR patterns. During the study period, trimethoprim remained the first-line therapy in the CMP; however, national Australian surveillance data reported increasing resistance rates of approximately 27%, prompting changes to the Australian Therapeutic UTI guidelines in March 2025, replacing trimethoprim with nitrofurantoin as the first-line treatment [62, 83]. While empirical treatment without microbiological confirmation may pose a potential risk for AMR, resistance patterns observed in the trial are consistent with ongoing developments in AMR trends rather than a deviation linked to the trial’s interventions [54, 62, 83].

The findings among patients who did not receive an antibiotic from the pharmacist should also be considered in the context of the natural course of uUTI symptoms. Previous literature suggests that spontaneous resolution uUTI symptoms (i.e., without antibiotic treatment) occurs in approximately 27–42% of patients who consult a GP [73–82]. In PATH-UTI, among the 796 patients who completed a follow-up survey without receiving an antibiotic from the pharmacist, 63.4% (505/796) reported complete symptom resolution by day 7. Among those reporting complete symptom resolution, 54.6% (276/505) also reported attending another healthcare professional, and 87.7% (242/276) indicated that they received antibiotic treatment from that healthcare professional. Therefore, symptom resolution in this group may have been partly attributable to subsequent care received outside the pharmacist-led service.

### Secondary outcomes: prescribing rates, adherence, referrals, and adverse events

Prescribing rates observed in this study are comparable to those reported in both pharmacy-based and physician-led models of care. The Australian Report on Antimicrobial Use and Resistance in Human Health (AURA) 2023 reported that antibiotics were prescribed in 90.8% of general practice consultations for UTIs, with trimethoprim used as first-line treatment in only 42% of cases [83]. While these prescribing rates are comparable, general practice consultations may include patients with greater clinical complexity and higher-acuity presentations than those eligible for management through the community pharmacy service [84, 85]. Previous pharmacy-based studies have reported prescribing rates between 80 to 97%, while physician-led trials have generally observed rates between 90 and 97% [73–77]. Higher prescribing rates across these studies are characteristic of empirical treatment models for uUTIs, where laboratory confirmation is not routinely required and treatment is initiated based on symptom presentation alone [76–82].

Self-reported adherence to antibiotic therapy was high, with 88.7% of patients reporting that they took their medication as prescribed. Adherence was highest among patients prescribed trimethoprim (94.0%), which may be attributed to its simpler once-daily dosing regimen [62]. In contrast, adherence was lower for nitrofurantoin (89.8%), which requires four-times-daily dosing, and cefalexin (91.3%), which is dosed twice daily [62]. This reduced adherence to nitrofurantoin may reflect the increased complexity of its four-times-daily dosing schedule. This has potential antimicrobial stewardship implications, as incomplete or inconsistent antibiotic use may contribute to treatment failure and recurrence [86]. These findings are consistent with international literature, which suggests that simpler dosing schedules are associated with improved adherence in antimicrobial therapy for uUTIs [86–88].

The referral rate of 6.9% was within the range reported in other studies conducted in Australia and the UK, which range from 3.7% to 16.7% depending on study design, population, and referral definitions [20, 27, 46, 89, 90]. The Queensland UTI Pharmacy Pilot (UTIPP-Q) reported a referral rate of 3.7% to GPs, while another Australian study reported a 5.2% referral rate [27, 90]. Referral rates reported in UK studies have ranged from 6.0% to 16.7%, placing PATH-UTI at the lower end of the UK-reported range [20, 89].

Whilst the referral rate in the PATH-UTI trial may seem modest, it could underestimate the ‘true’ referral rate due to informal pre-screening practices adopted by some pharmacists. Anecdotal evidence from PCF visits revealed that pharmacists occasionally conducted informal pre-screening prior to initiating a formal consultation. Patients deemed ineligible (e.g., due to red-flag symptoms) could have been advised to seek medical care and not entered into MedAdvisor, meaning these encounters were not formally captured as referrals. Although such informal pre-screening poses challenges for accurately quantifying total referral rates, it may also reflect pragmatic clinical judgement exercised by pharmacists [91].

The rate of patient-reported adverse events was low. The majority were gastrointestinal and transient in nature (61.5%), which aligns with the existing literature, as gastrointestinal symptoms are commonly reported with antimicrobial prescribing [92]. Comparison with published literature and clinical databases suggests that the overall incidence of adverse effects observed in PATH-UTI was low and consistent with similar trials. For example, comparable rates were observed in a study by Beahm et al., in which 7.2% (n = 54) of patients reported adverse events during treatment, with gastrointestinal adverse events (59.3%), secondary vaginal infections (14.8%) and headache (11.1%) among the most common [24]. Notably, Beahm et al. found no statistically significant differences in adverse event rates between patients treated by pharmacists and those managed by GPs [24]. In the PATH-UTI trial, most adverse events were reported in patients prescribed trimethoprim (88.1%), which is consistent with trimethoprim being the most commonly prescribed antibiotic by pharmacists (95.5%) [62]. However, when adverse events were examined using the number of patients prescribed each antibiotic as the denominator, the reported adverse event rate was higher for nitrofurantoin (12.9%). This is consistent with existing literature recognising that nitrofurantoin may be associated with a higher frequency of adverse effects than some alternative treatments [93]. Interpretation should remain cautious due to the smaller number of patients prescribed nitrofurantoin and cefalexin, though no serious adverse events were reported for either.

The findings presented in this paper are limited to pharmacy management data gathered through the pharmacy consultation and patient survey. As linked administrative health data were not included in the present analysis, we were unable to determine whether patients requiring repeat treatment were subsequently referred to or reviewed by a GP for suspected recurrent infection. Complementary analyses using linked administrative health datasets have been undertaken to evaluate broader safety and effectiveness outcomes, including repeat presentations, subsequent healthcare utilisation, hospitalisations, ED presentations, GP services captured through the Medicare Benefits Schedule (MBS), and Pharmaceutical Benefits Scheme (PBS) utilisation [54].

### Strengths and weaknesses

This study has several strengths. First, it included a large and diverse sample of more than 18,000 consultations across metropolitan, regional, and rural settings, representing one of the largest evaluations of pharmacist prescribing to date. Secondly, the follow-up rate of 81.0% is substantially higher than previous trials conducted in Australia, Canada, NZ and the UK, where rates as low as 28.9% have been reported [24, 27, 69, 94]. Thirdly, the methodology included the use of PCFs, which was central to the implementation strategy.

The limitations included the absence of a comparison group, which limits the capacity to compare the findings and individualise the effect of the intervention and increases the risk of internal and external bias. While a randomised controlled trial (RCT) would have provided greater internal validity, this design was not feasible in a pragmatic, real-world implementation [95]. To mitigate these concerns, the results were compared against existing literature on pharmacist- and physician-led uUTI management, an accepted approach for reducing bias in non-comparator studies [96]. Lastly, reliance on self-reported symptom outcomes introduces the possibility of recall or reporting bias. However, high follow-up rates likely reduce this risk, and self-reported symptom resolution has been accepted as a valid outcome measure in similar studies [24, 27].

## Conclusion

Overall, the findings of this study contribute to the growing body of real-world evidence supporting pharmacist prescribing for uUTIs when conducted under a structured CMP and appropriate regulatory authority. The high rate of symptom resolution, low rate of patient-reported adverse events, and strong adherence to clinical protocols, including appropriate antibiotic selection and referral pathways, suggest that community pharmacists can contribute to the safe and effective management of uUTIs in primary care. By examining symptom resolution and adverse events by antibiotic type, this study provides further insight into the effectiveness and safety of pharmacist prescribing in routine practice. Pharmacist prescribing for uUTIs has the potential to improve timely access to care and help manage demand on general practice and other healthcare services. However, this study focused on pharmacist management practices and patient-reported clinical outcomes. Broader system-level impacts, including outcomes assessed using linked administrative health datasets, have been reported elsewhere [54].

## Supplementary Information

Below is the link to the electronic supplementary material.

Supplementary file1 (PDF 281 kb)

Supplementary file2 (PDF 597 kb)

Supplementary file3 (PDF 156 kb)

Supplementary file4 (PDF 276 kb)

Supplementary file5 (PDF 187 kb)

## Data Availability

The data that support the findings of this study are available from the corresponding author, MB, upon reasonable request.
