# Supplementary material for: Pathway to Access UTI Management (PATH-UTI): evaluating pharmacist prescribing and management practices for uncomplicated urinary tract infections in community pharmacy in New South Wales and the Australian Capital Territory, Australia
Source: Int J Clin Pharm. 2026 Sep 23;48(6):2490–503. doi: 10.1007/s11096-026-02226-9 (PMC13731454; doi:10.1007/s11096-026-02226-9)
Supplement: Supplementary file 1 [file 11096_2026_2226_MOESM1_ESM.pdf]

POISONS AND THERAPEUTIC GOODS ACT 1966

Section 10 Poisons and Therapeutic Goods Act 1966

Clauses 170 and 171 of the Poisons and Therapeutic Goods Regulation 2008

AUTHORITY

I, Dr Kerry Chant, Chief Health Officer, a duly appointed delegate of the Secretary, NSW Health, pursuant to clauses 53, 170, and 171 of the Poisons and Therapeutic Goods Regulation 2008 for the purpose of section 10 of the Poisons and Therapeutic Goods Act 1966, hereby:

1. cancel the instrument signed 19 April 2023; and
2. make this instrument.

Dr Kerry Chant

Chief Health Officer

Dated: 04/07/2023

Authority – Supply of specified restricted substances by pharmacists

---

1) Authorisation

This instrument authorises an 'approved pharmacist' to supply to an 'applicable patient' a restricted substance listed in clause 2 otherwise than on prescription subject to the conditions in clause 3 of this instrument for the purposes of the 'clinical trial'.

2) Restricted substance to which this instrument applies

This instrument applies to oral forms of:

a. trimethoprim
b. nitrofurantoin
c. cefalexin

3) Conditions — Limitation on supply

An approved pharmacist may supply the restricted substance listed in clause 2, subject to the conditions that:

a. The pharmacist must only supply nitrofurantoin if trimethoprim is unavailable or inappropriate for treatment of the particular patient.

b. The pharmacist must only supply cefalexin if both trimethoprim and nitrofurantoin are unavailable or inappropriate for treatment of the particular patient.

c. The pharmacist must not sell a medicine specified in clause 2 in a quantity that exceeds the smallest available size of the manufacturer's pack of the medicine.

d. The pharmacist must comply with the 'Management Protocols', including the requirement that the pharmacist makes a record in MedAdvisor pharmacy software, or an approved system by the Ministry of Health, regarding the supply.

e. The pharmacist must make and keep a clinical record of the consultation for 7 years (at the pharmacy where the patient consultation occurred) that contains:

  - sufficient information to identify the patient
  - the date of the treatment
  - the name of the pharmacist who undertook the consultation
  - any information known to the pharmacist that is relevant to the patient's diagnosis or treatment (for example, information concerning the patient's medical history)
  - any clinical opinion reached by the pharmacist
  - actions taken by the pharmacist
  - particulars of any medication supplied for the patient (such as form, strength and amount)
  - notes as to information or advice given to the patient in relation to any treatment proposed by the pharmacist who is treating the patient
  - any consent given by a patient to the treatment proposed.

f. The pharmacist must share a record of the supply with the patient's usual treating medical practitioner or medical practice, where the patient has one, following consent by the patient.

g. The pharmacist must consent to participate in the clinical trial and its evaluation, including by sharing records of applicable patients with the University of Newcastle.

h. The pharmacist must comply with the AHPRA & National Boards Code of Conduct; and the expected standards of ethical behaviour of pharmacists towards individuals, the community and society.

4) Publication

This instrument will be published on the NSW Health website.

5) Definitions

In this instrument:

- An 'applicable patient' means a female patient 18 years of age or over and up to and including aged 65 years.
- An 'approved pharmacist' means a pharmacist holding general registration under the *Health Practitioner Regulation National Law* and who is employed or engaged in an 'approved pharmacy' who has successfully completed the following training:

  - Australasian College of Pharmacy Uncomplicated Cystitis Treatment – Pharmacist Training; or
  - Pharmaceutical Society of Australia Managing uncomplicated cystitis; and
  - Training module(s) that have been approved by the Chief Health Officer for the purposes of the clinical trial.
- An 'approved pharmacy' means a pharmacy or class of pharmacies approved in writing by the Chief Health Officer which:
  - offers applicable patients the services specified in this authorisation at all opening hours of the pharmacy; and
  - has a service room, consulting room, or area consistent with the following:
    - the room or area is not to be used as a dispensary, storeroom, staff room or retail area,
    - fully enclosed and provides adequate privacy (a divider or curtain in a dispensary, storeroom, staff room or retail area is not acceptable),
    - has adequate lighting,
    - is maintained at a comfortable ambient temperature,
    - has a hand sanitisation facility,
    - has ready access to a hand washing facility, and
    - has sufficient floor area, clear of equipment and furniture, to accommodate the person receiving the consultation and an accompanying person, and to allow the pharmacist adequate space to manoeuvre.
- 'Management protocols' means the protocols established for use by pharmacists in the clinical trial.
- The 'clinical trial' means the trial put in place by the University of Newcastle on behalf of the Ministry of Health regarding the management of urinary tract infections by community pharmacists.
- A 'pharmacy' has the same meaning as in the Health Practitioner Regulation National Law.

6) Commencement

This authority commences on publication.

7) Cancellation

This authority is cancelled on 18 July 2024, unless earlier cancelled.
