# Supplementary material for: Pathway to Access UTI Management (PATH-UTI): evaluating pharmacist prescribing and management practices for uncomplicated urinary tract infections in community pharmacy in New South Wales and the Australian Capital Territory, Australia
Source: Int J Clin Pharm. 2026 Sep 23;48(6):2490–503. doi: 10.1007/s11096-026-02226-9 (PMC13731454; doi:10.1007/s11096-026-02226-9)
Supplement: Supplementary file 2 [file 11096_2026_2226_MOESM2_ESM.pdf]

Human Research Ethics

Participant Information Statement (Pharmacy - NSW)

Clinical Research

THE UNIVERSITY OF NEWCASTLE AUSTRALIA

Dr Sarah Dineen-Griffin
College of Health, Medicine and Wellbeing
University of Newcastle
Phone number: 02 4055 0155
Email: NSWPharmacyTrial@newcastle.edu.au

Regarding Research Project: **NSW Government-Sponsored Clinical Trial: Management of Urinary Tract Infections by Community Pharmacists (Intervention Study) – Information for Pharmacies - NSW**

Dear Pharmacy Owner,

Your pharmacy is invited to participate in the research project which is being conducted by a consortium of researchers led by the University of Newcastle, The George Institute for Global Health (TGI) and the Hunter Medical Research Institute. Please note each pharmacist working in your pharmacy has the option to voluntarily provide consent for their participation. A separate consent form is available to be signed. This project has been funded by the NSW Government. The information below provides details of the study and how your pharmacy can participate if you choose to do so.

1. What is the research study about?

The aim of this overall research is to evaluate the implementation, clinical and economic impact of a service model (intervention) delivered by community pharmacists to women aged between 18 years and 65 years in NSW and selected pharmacies in the ACT. Your pharmacy is being invited to participate in a trial of the new service over 10 months.

2. Who is conducting the research?

This research is being conducted under the direction of Dr Sarah Dineen-Griffin from the University of Newcastle's School of Biomedical Sciences and Pharmacy. The evaluation is being led by The George Institute for Global Health in partnership with University of Newcastle and the Hunter Medical Research Institute. The research personnel involved, their affiliations and roles are identified below, under the heading 'Research Team'.

3. Who can participate in the research?

This research study is proactively recruiting pharmacies that meet the criteria outlined in the NSW Health Authority for pharmacies to participate (please see attachment). For your information, we are highlighting the eligibility criteria for an approved pharmacy to participate in the study:

"An 'approved pharmacy' means a pharmacy or class of pharmacies, approved in writing by the Chief Health Officer, which has a service room, consulting room, or area consistent with the following:
o the room or area is not to be used as a dispensary, storeroom, staff room or retail area,
o fully enclosed and provides adequate privacy (a divider or curtain in a dispensary, storeroom, staff room or retail area is not acceptable),
o has adequate lighting,
o is maintained at a comfortable ambient temperature,
o has a hand sanitisation facility,
o has ready access to a hand washing facility, and
o has sufficient floor area, clear of equipment and furniture, to accommodate the person receiving the consultation and an accompanying person, and to allow the pharmacist adequate space to manoeuvre."

Pharmacies must have access to MedAdvisor to complete clinical record keeping for the purposes of the clinical trial assessment.
We are also highlighting the eligibility criteria for an approved pharmacist to participate in the study, which includes that the pharmacist must be:

"employed or engaged in an 'approved pharmacy' who has successfully completed the following training:

- Australasian College of Pharmacy Uncomplicated Cystitis Treatment – Pharmacist Training; or
- Pharmaceutical Society of Australia Managing uncomplicated cystitis; and
- A series of study training module(s) that have been approved by the Chief Health Officer for the purposes of the clinical trial."

A pharmacist is eligible to participate if they hold general registration as a pharmacist with the Australian Health Practitioner Regulation Agency (AHPRA). Pharmacists with provisional registration (intern pharmacists) and pharmacists with conditions on their registration are not eligible to participate in the trial.

If your pharmacy (or your pharmacists) does not meet these eligibility criteria, your pharmacy will not be able to participate in the study.

The pharmacy must have at least one eligible pharmacist who is willing to provide their voluntary consent to participate, for the pharmacy to be eligible. I understand that it is my responsibility as a pharmacy owner, that there is always a pharmacist that has consented to participate in the trial and is available to deliver the service during all opening hours of the pharmacy.

It is the pharmacy owner's responsibility to ensure that only pharmacists that have provided voluntary consent and meet the NSW Health Authority requirements to provide the service.

4. What does participation involve?

If you agree for your pharmacy to participate, and you have pharmacists who meet the eligibility criteria, your pharmacists will be asked to offer a UTI service (approximately 10 minutes per consult) to females aged 18-65 years presenting to your community pharmacy with urinary symptoms. Your pharmacists will also be asked to complete specific training and document data according to an agreed protocol over approximately 10 months. Please refer to the attached NSW Health Authority. In addition to providing the service to the patient, the researchers will visit/contact your pharmacy to discuss the implementation process in your pharmacy throughout the trial period. The research team anticipate at least monthly touchpoints per pharmacy for support during the trial period, however in some instances, this may be more than monthly. If your pharmacy is deemed to need more support, you will be provided with additional resources, as required. Support may involve a member of the research team visiting or calling the pharmacy, completing checklists to capture barriers and facilitators to service delivery and implementation, and may provide the pharmacy with strategies and solutions to overcome the identified practice barriers. This support may be provided face to face, by phone or by Zoom.

Your pharmacists may also be contacted by the research team with an invitation to participate in an interview.

5. Do you have to take part in this research study?

No. Participation in this study is voluntary. If you do not want your pharmacy to take part, you do not have to. And, if you do agree to participate, you have the option of withdrawing your pharmacy at any time, we will thank you for your time.

If a specific pharmacist in your pharmacy does not wish to participate, the pharmacist's decision regarding participation should not impact on their relationship with you as their employer.

It is not necessary for all pharmacists that work in the pharmacy to provide the service.

6. What is the benefit of participating in this research study?

We cannot guarantee or promise that you will receive any benefits from this research; however, findings of this study may be of potential benefit to you in the future. This may include the researchers providing evidence to the NSW Government on the value of pharmacies and pharmacists as part of the national health reform agenda and expanding the scope of practice for community pharmacy.

A payment of $20 per patient consultation will be made to the pharmacy, irrespective of the consult outcome. Data on these consultations will be made available to the research team, through the MedAdvisor software, or any other approved system by the Ministry of Health.

7. Are there any risks associated with your participation in this research?

There are minimal risks associated with this research; however unexpected risks may occur. Inconvenience may occur due to the time taken to complete consultations with patients, and training. Pharmaceutical Defence Limited (PDL) is the major provider of professional indemnity and will cover pharmacists as part of their normal indemnity for delivery of this service, following notification of participation. Guild Business will cover the liability from the perspective of the pharmacy premises.

You can also contact the Chief Investigator, Dr Sarah Dineen-Griffin on 02 4055 0155 or NSWPharmacyTrial@newcastle.edu.au, should you need support regarding any of the issues raised in the research activities.

8. How will your privacy be protected?

Any information obtained in connection with this study that can identify your pharmacy will remain confidential. An ID code will be assigned to your pharmacy through MedAdvisor. Data will be retained securely for a minimum period of fifteen years from the completion of the research project. Information will be managed and stored in accordance with the University's Research Data and Materials Management Guideline or any successor Guideline as well as any other applicable University of Newcastle or TGI policy provisions.

9. How will information collected by the research team be used?

Findings will be reported to the NSW Government, in peer-reviewed publications and conference presentations. Individual participants will not be identifiable in any of the publications generated. Non-identifiable data may be shared with other parties as part of a peer-review process to verify the robustness and integrity of the study, or to contribute to further research and public knowledge. If you would like a copy of the summary of the results, please provide your email address, or mailing address, on the consent form so a summary of the results can be shared with you. The data generated will be used as part of a PhD thesis by Simone Diamandis, Mitchell Budden and Victoria Chisari (under the supervision of Dr Sarah Dineen-Griffin).

10. What you need to do to provide consent to participate?

Read this Information Statement in its entirety and be sure you understand all the information provided before agreeing for your pharmacy to participate. If there is anything you do not understand, or if you have questions, contact the Chief Investigator, Dr Sarah Dineen-Griffin on 02 4055 0155 or NSWPharmacyTrial@newcastle.edu.au for more information. If you decide for your pharmacy to participate, please complete and sign the Consent Form via the DocuSign link which will be shared with you by the research team. If you do not respond within one week of the initial invitation, a second invitation will be sent. If you do not respond within 3 working days of the second invitation, we will presume that your pharmacy does not want to participate. We will not contact you any further after this time.

11. What if you want to withdraw from the research study?

Your pharmacy may withdraw from the study at any time, without providing a reason, by contacting the Chief Investigator, Dr Sarah Dineen-Griffin on 02 4055 0155 or NSWPharmacyTrial@newcastle.edu.au. Once collected, data cannot be withdrawn from the study. The decision for your pharmacy not to participate or to withdraw from the study will not affect your relationship with the University of Newcastle or any of the other organisations involved in this research.

12. Do you need more information?

If you would like more information about this research project and your potential involvement, please contact the Chief Investigator, Dr Sarah Dineen-Griffin on 02 4055 0155 or NSWPharmacyTrial@newcastle.edu.au.

13. Research Team

- Dr Sarah Dineen-Griffin, The University of Newcastle (Chief Investigator)
- Emeritus Professor Shalom I Benrimoj, The University of Sydney (Co-Investigator)
- Professor David Peiris, The George Institute for Global Health (Co-Investigator)
- Dr Belinda Ford, The George Institute for Global Health (Co-Investigator)
- Dr Gill Schierhout, The George Institute for Global Health (Co-Investigator)
- Dr Anna Campain, The George Institute for Global Health (Co-Investigator)
- Associate Professor Kris Rogers, University of Technology Sydney (Co-Investigator)
- Associate Professor Penny Reeves, Hunter Medical Research Institute (Co-Investigator)
- Emeritus Professor Julie Byles AO, Hunter Medical Research Institute (Co-Investigator)
- Dr Indy Sandaradura, Centre for Infectious Diseases and Microbiology (Co-Investigator)
- Dr Leanne Holt, Macquarie University (Co-Investigator)
- Dr Kylie Gwynne, Macquarie University (Co-Investigator)
- Professor Kylie Williams, University of Technology Sydney (Co-Investigator)
- Dr Helen Benson, University of Technology Sydney (Co-Investigator)
- Associate Professor John Rae, Charles Sturt University (Co-Investigator)
- Ms Anna Barwick, University of New England (Co-Investigator)
- Dr Joanna Moullin, Curtin University (Co-Investigator)
- Ms Jan Donovan, Consumer representative (Co-Investigator)
- Simone Diamandis, The University of Newcastle (PhD Student) – the data will contribute to her PhD thesis.
- Mitchell Budden, The University of Newcastle (PhD Student) – the data will contribute to his PhD thesis.
- Victoria Chisari, The University of Technology Sydney (PhD Student) – the data will contribute to her PhD thesis.

Thank you for your time and consideration of this information.

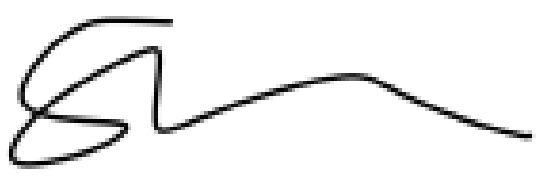

Dr Sarah Dineen-Griffin
Chief Investigator

University of Newcastle

Concerns or complaints about this research

This project has been approved by the University's Human Research Ethics Committee, Approval No. H-[2023-0119]. If you have concerns about your rights as a participant in this research, or if you have a complaint about the manner in which the research is conducted, please contact the Chief Investigator, Dr Sarah Dineen-Griffin on 02 4055 0155 or NSWPharmacyTrial@newcastle.edu.au. If you would prefer to contact someone independent of the research project, you can forward your concerns to:

Human Research Ethics Officer
Research and Innovation Services
University of Newcastle
University Drive
Callaghan NSW 2308, Australia
Phone: (02) 4921 6333
Email: Human-Ethics@newcastle.edu.au

Human Research Ethics

Research Participant Consent Form (Pharmacy - NSW)

THE UNIVERSITY OF NEWCASTLE AUSTRALIA

Research Project Information

NSW Government-Sponsored Clinical Trial: Management of Urinary Tract Infections by Community Pharmacists (Intervention Study) – Information for Pharmacy - NSW

Dr Sarah Dineen-Griffin
College of Health, Medicine and Wellbeing
University of Newcastle
Phone number: 02 4055 0155
Email: NSWPharmacyTrial@newcastle.edu.au

Participant Consent

I agree for my pharmacy to participate in the above research project and give my consent freely.

I confirm that my pharmacy meets all study requirements and the requirements of the NSW Health Authority.

I understand that it is my responsibility as a pharmacy owner, that there is always a pharmacist that has consented to participate in the trial and is available to deliver the service during all opening hours of the pharmacy.

I understand that my pharmacy must have access to MedAdvisor to complete clinical record keeping for the purposes of the clinical trial assessment.

I understand that the project will be conducted as described in the Participant Information Statement, a copy of which I have read and retained.

I understand that my pharmacy can withdraw from the project as explained in the Participant Information Statement, and I do not have to give any reason for my pharmacy's decision to withdraw.

I consent to my pharmacy participating in a 10 month study which will involve an approved pharmacist delivering a new service to patients and documenting data.

I understand that any pharmacy information will remain confidential to the researchers, except as required by law.

I understand that an ID code will be assigned to my pharmacy through MedAdvisor. Any information obtained in connection with this study that can identify your pharmacy will remain confidential, except as required by law.

I consent for MedAdvisor software, or an approved system by the Ministry of Health, to provide patient consultation and pharmacy data to the research team.

I consent to my pharmacy contact details being shared on an online public register for consumers to identify pharmacies participating in the trial and offering the service.

I consent to the research team informing Guild Insurance, or any other insurer, of my pharmacy's participation in the study.

I understand that my pharmacy will be visited/contacted by the research team at least once throughout the main study to discuss implementation of the service in my pharmacy.

I have had the opportunity to have questions answered to my satisfaction.

Human Research Ethics

Research Participant Consent Form (Pharmacy - NSW)

THE UNIVERSITY OF NEWCASTLE AUSTRALIA

Participant Details

Pharmacy name (please print)	
Pharmacy owner name (please print)	
Signature	
Email address	
Phone number	
Date	
Pharmacy insurance provider name	

Pharmacist details

You are asked to provide the researchers with individual names and contact details of pharmacists in your pharmacy who have voluntarily indicated that they wish to deliver the service in your pharmacy, noting they will be required to provide individual signed consent to participate. Each pharmacist will receive a Participant Information Statement and consent form directly by email from the research team. Please ensure you have the permission of the pharmacist, before providing their details below.

Pharmacist first name (please print)	Pharmacist last name (please print)	Pharmacist individual email address

Receiving Research Results

I would like the research team to send me a summary of the research results once available.

☐ Yes

☐ No

If yes, I would like a copy sent to me via:

☐ the email address noted on this form

☐ another address (please specify): *[insert address]*

Human Research Ethics

Participant Information Statement (Pharmacy - ACT)

Clinical Research

THE UNIVERSITY OF NEWCASTLE AUSTRALIA

Dr Sarah Dineen-Griffin
College of Health, Medicine and Wellbeing
University of Newcastle
Phone number: 02 4055 0155
Email: NSWPharmacyTrial@newcastle.edu.au

Regarding Research Project: **NSW Government-Sponsored Clinical Trial: Management of Urinary Tract Infections by Community Pharmacists (Intervention Study) – Information for Pharmacies - ACT**

Dear Pharmacy Owner,

Your pharmacy is invited to participate in the research project which is being conducted by a consortium of researchers led by the University of Newcastle, The George Institute for Global Health (TGI) and the Hunter Medical Research Institute. Please note each pharmacist working in your pharmacy has the option to voluntarily provide consent for their participation. A separate consent form is available to be signed. This project has been funded by the NSW Government. The information below provides details of the study and how your pharmacy can participate if you choose to do so.

1. What is the research study about?

The aim of this overall research is to evaluate the implementation, clinical and economic impact of a service model (intervention) delivered by community pharmacists to women aged between 18 years and 65 years in NSW and selected pharmacies in the ACT. Your pharmacy is being invited to participate in a trial of the new service over 10 months.

2. Who is conducting the research?

This research is being conducted under the direction of Dr Sarah Dineen-Griffin from the University of Newcastle's School of Biomedical Sciences and Pharmacy. The evaluation is being led by The George Institute for Global Health in partnership with University of Newcastle and the Hunter Medical Research Institute. The research personnel involved, their affiliations and roles are identified below, under the heading 'Research Team'.

3. Who can participate in the research?

Pharmacies in the ACT will be granted a licence by ACT Health to participate in the trial and provide the UTI service (please find attached a PDF copy of the licence application). For your information, we are highlighting the eligibility criteria for an approved pharmacy to participate in the study:

"An 'approved pharmacy' means a pharmacy or class of pharmacies, approved in writing by the Chief Health Officer, which has a service room, consulting room, or area consistent with the following:
o the room or area is not to be used as a dispensary, storeroom, staff room or retail area,
o fully enclosed and provides adequate privacy (a divider or curtain in a dispensary, storeroom, staff room or retail area is not acceptable),
o has adequate lighting,
o is maintained at a comfortable ambient temperature,
o has a hand sanitisation facility,
o has ready access to a hand washing facility, and
o has sufficient floor area, clear of equipment and furniture, to accommodate the person receiving the consultation and an accompanying person, and to allow the pharmacist adequate space to manoeuvre."

Pharmacies must have access to MedAdvisor to complete clinical record keeping for the purposes of the clinical trial assessment.
We are also highlighting the eligibility criteria for an approved pharmacist to participate in the study, which includes that the pharmacist must be:

"employed or engaged in an 'approved pharmacy' who has successfully completed the following training:

- Australasian College of Pharmacy Uncomplicated Cystitis Treatment – Pharmacist Training; or
- Pharmaceutical Society of Australia Managing uncomplicated cystitis; and
- A series of study training module(s) that have been approved by the Chief Health Officer for the purposes of the clinical trial."

A pharmacist is eligible to participate if they hold general registration as a pharmacist with the Australian Health Practitioner Regulation Agency (AHPRA). Pharmacists with provisional registration (intern pharmacists) and pharmacists with conditions on their registration are not eligible to participate in the trial.

If your pharmacy (or your pharmacists) does not meet these eligibility criteria, your pharmacy will not be able to participate in the study.

The pharmacy must have at least one eligible pharmacist who is willing to provide their voluntary consent to participate, for the pharmacy to be eligible. I understand that it is my responsibility as a pharmacy owner, that there is always a pharmacist that has consented to participate in the trial and is available to deliver the service during all opening hours of the pharmacy.

It is the pharmacy owner's responsibility to ensure that only pharmacists that have provided voluntary consent and meet the requirements set out by ACT Health to provide the service.

4. What does participation involve?

If you agree for your pharmacy to participate, and you have pharmacists who meet the eligibility criteria, your pharmacists will be asked to offer a UTI service (approximately 10 minutes per consult) to females aged 18-65 years presenting to your community pharmacy with urinary symptoms. Your pharmacists will also be asked to complete specific training and document data according to an agreed protocol over approximately 12 months. In addition to providing the service to the patient, the researchers will visit/contact your pharmacy to discuss the implementation process in your pharmacy throughout this trial period. The research team anticipate at least monthly touchpoints per pharmacy for support during the trial period, however in some instances, this may be more than monthly. If your pharmacy is deemed to need more support, you will be provided with additional resources, as required. Support may involve a member of the research team visiting or calling the pharmacy, completing checklists to capture barriers and facilitators to service delivery and implementation, and may provide the pharmacy with strategies and solutions to overcome the identified practice barriers. This support may be provided face to face, by phone or by Zoom.

Your pharmacists may also be contacted by the research team with an invitation to participate in an interview.

5. Do you have to take part in this research study?

No. Participation in this study is voluntary. If you do not want your pharmacy to take part, you do not have to. And, if you do agree to participate, you have the option of withdrawing your pharmacy at any time, we will thank you for your time.

If a specific pharmacist in your pharmacy does not wish to participate, the pharmacist's decision regarding participation should not impact on their relationship with you as their employer.

It is not necessary for all pharmacists that work in the pharmacy to provide the service.

6. What is the benefit of participating in this research study?

We cannot guarantee or promise that you will receive any benefits from this research; however, findings of this study may be of potential benefit to you in the future. This may include the researchers providing evidence to the NSW Government on the value of pharmacies and pharmacists as part of the national health reform agenda and expanding the scope of practice for community pharmacy.

A consultation fee will be paid for by the patient, irrespective of the consult outcome. Data on these consultations will be made available to the research team, through the MedAdvisor software, or any other approved system by the Ministry of Health.

7. Are there any risks associated with your participation in this research?

There are minimal risks associated with this research; however unexpected risks may occur. Inconvenience may occur due to the time taken to complete consultations with patients, and training.

Pharmaceutical Defence Limited (PDL) will cover pharmacists as part of their normal indemnity for delivery of this service following notification of participation. Guild Business will cover the liability from the perspective of the pharmacy premises.

You can also contact the Chief Investigator, Dr Sarah Dineen-Griffin on 02 4055 0155 or NSWPharmacyTrial@newcastle.edu.au, should you need support regarding any of the issues raised in the research activities.

8. How will your privacy be protected?

Any information obtained in connection with this study that can identify your pharmacy will remain confidential. An ID code will be assigned to your pharmacy through MedAdvisor. Data will be retained securely for a minimum period of fifteen years from the completion of the research project. Information will be managed and stored in accordance with the University's Research Data and Materials Management Guideline or any successor Guideline as well as any other applicable University of Newcastle or TGI policy provisions.

9. How will information collected by the research team be used?

Findings will be reported to the NSW Government, in peer-reviewed publications and conference presentations. Individual participants will not be identifiable in any of the publications generated. Non-identifiable data may be shared with other parties as part of a peer-review process to verify the robustness and integrity of the study, or to contribute to further research and public knowledge. If you would like a copy of the summary of the results, please provide your email address, or mailing address, on the consent form so a summary of the results can be shared with you. The data generated will be used as part of a PhD thesis by Simone Diamandis, Mitchell Budden and Victoria Chisari (under the supervision of Dr Sarah Dineen-Griffin)..

10. What you need to do to provide consent to participate?

Read this Information Statement in its entirety and be sure you understand all the information provided before agreeing for your pharmacy to participate. If there is anything you do not understand, or if you have questions, contact the Chief Investigator, Dr Sarah Dineen-Griffin on 02 4055 0155 or NSWPharmacyTrial@newcastle.edu.au for more information. If you decide for your pharmacy to participate, please complete and sign the Consent Form via the DocuSign link which will be shared with you by the research team. If you do not respond within one week of the initial invitation, a second invitation will be sent. If you do not respond within 3 working days of the second invitation, we will presume that your pharmacy does not want to participate. We will not contact you any further after this time.

11. What if you want to withdraw from the research study?

Your pharmacy may withdraw from the study at any time, without providing a reason, by contacting the Chief Investigator, Dr Sarah Dineen-Griffin on 02 4055 0155 or NSWPharmacyTrial@newcastle.edu.au. Once collected, data cannot be withdrawn from the study. The decision for your pharmacy not to participate or to withdraw from the study will not affect your relationship with the University of Newcastle or any of the other organisations involved in this research.

12. Do you need more information?

If you would like more information about this research project and your potential involvement, please contact the Chief Investigator, Dr Sarah Dineen-Griffin on 02 4055 0155 or NSWPharmacyTrial@newcastle.edu.au.

13. Research Team

- Dr Sarah Dineen-Griffin, The University of Newcastle (Chief Investigator)
- Emeritus Professor Shalom I Benrimoj, The University of Sydney (Co-Investigator)
- Professor David Peiris, The George Institute for Global Health (Co-Investigator)
- Dr Belinda Ford, The George Institute for Global Health (Co-Investigator)
- Dr Gill Schierhout, The George Institute for Global Health (Co-Investigator)
- Dr Anna Campain, The George Institute for Global Health (Co-Investigator)
- Associate Professor Kris Rogers, University of Technology Sydney (Co-Investigator)
- Associate Professor Penny Reeves, Hunter Medical Research Institute (Co-Investigator)
- Emeritus Professor Julie Byles AO, Hunter Medical Research Institute (Co-Investigator)
- Dr Indy Sandaradura, Centre for Infectious Diseases and Microbiology (Co-Investigator)
- Dr Leanne Holt, Macquarie University (Co-Investigator)
- Dr Kylie Gwynne, Macquarie University (Co-Investigator)
- Professor Kylie Williams, University of Technology Sydney (Co-Investigator)
- Dr Helen Benson, University of Technology Sydney (Co-Investigator)
- Associate Professor John Rae, Charles Sturt University (Co-Investigator)
- Ms Anna Barwick, University of New England (Co-Investigator)
- Dr Joanna Moullin, Curtin University (Co-Investigator)
- Ms Jan Donovan, Consumer representative (Co-Investigator)
- Simone Diamandis, The University of Newcastle (PhD Student) – the data will contribute to her PhD thesis.
- Mitchell Budden, The University of Newcastle (PhD Student) – the data will contribute to his PhD thesis.
- Victoria Chisari, The University of Technology Sydney (PhD Student) – the data will contribute to her PhD thesis.

Thank you for your time and consideration of this information.

Dr Sarah Dineen-Griffin
Chief Investigator
University of Newcastle

Concerns or complaints about this research

This project has been approved by the University's Human Research Ethics Committee, Approval No. H-[2023-0119]. If you have concerns about your rights as a participant in this research, or if you have a complaint about the manner in which the research is conducted, please contact the Chief Investigator, Dr Sarah Dineen-Griffin on 02 4055 0155 or NSWPharmacyTrial@newcastle.edu.au. If you would prefer to contact someone independent of the research project, you can forward your concerns to:

Human Research Ethics Officer
Research and Innovation Services
University of Newcastle
University Drive
Callaghan NSW 2308, Australia
Phone: (02) 4921 6333
Email: Human-Ethics@newcastle.edu.au

Human Research Ethics

Research Participant Consent Form (Pharmacy - ACT)

Research Project Information

NSW Government-Sponsored Clinical Trial: Management of Urinary Tract Infections by Community Pharmacists (Intervention Study) – Information for Pharmacy - ACT

Dr Sarah Dineen-Griffin
College of Health, Medicine and Wellbeing
University of Newcastle
Phone number: 02 4055 0155
Email: NSWPharmacyTrial@newcastle.edu.au

Participant Consent

I agree for my pharmacy to participate in the above research project and give my consent freely.

I confirm that my pharmacy meets all study requirements and the requirements of the ACT Health Licence.

I understand that it is my responsibility as a pharmacy owner, that there is always a pharmacist that has consented to participate in the trial and is available to deliver the service during all opening hours of the pharmacy.

I understand that my pharmacy must have access to MedAdvisor to complete clinical record keeping for the purposes of the clinical trial assessment.

I understand that the project will be conducted as described in the Participant Information Statement, a copy of which I have read and retained.

I understand that my pharmacy can withdraw from the project as explained in the Participant Information Statement, and I do not have to give any reason for my pharmacy's decision to withdraw.

I consent to my pharmacy participating in a 10 month study which will involve an approved pharmacist delivering a new service to patients and documenting data.

I understand that any pharmacy information will remain confidential to the researchers, except as required by law.

I understand that an ID code will be assigned to my pharmacy through MedAdvisor. Any information obtained in connection with this study that can identify your pharmacy will remain confidential, except as required by law.

I consent for MedAdvisor software, or an approved system by the Ministry of Health, to provide patient consultation and pharmacy data to the research team.

I consent to my pharmacy contact details being shared on an online public register for consumers to identify pharmacies participating in the trial and offering the service.

I consent to the research team informing Guild Insurance, or any other insurer, of my pharmacy's participation in the study.

I understand that my pharmacy will be visited/contacted by the research team at least once throughout the main study to discuss implementation of the service in my pharmacy.

I have had the opportunity to have questions answered to my satisfaction.

Human Research Ethics

Research Participant Consent Form (Pharmacy - ACT)

THE UNIVERSITY OF NEWCASTLE AUSTRALIA

Participant Details

Pharmacy name (please print)	
Pharmacy owner name (please print)	
Signature	
Email address	
Phone number	
Date	
Pharmacy insurance provider name	

Pharmacist details

You are asked to provide the researchers with individual names and contact details of pharmacists in your pharmacy who have voluntarily indicated that they wish to deliver the service in your pharmacy, noting they will be required to provide individual signed consent to participate. Each pharmacist will receive a Participant Information Statement and consent form directly by email from the research team. Please ensure you have the permission of the pharmacist, before providing their details below.

Pharmacist first name (please print)	Pharmacist last name (please print)	Pharmacist individual email address

Receiving Research Results

I would like the research team to send me a summary of the research results once available.

☐ Yes

☐ No

If yes, I would like a copy sent to me via:

☐ the email address noted on this form

☐ another address (please specify): *[insert address]*

Human Research Ethics

Participant Information Statement (Pharmacist - NSW)

Clinical Research

Dr Sarah Dineen-Griffin
College of Health, Medicine and Wellbeing
University of Newcastle
Phone number: 02 4055 0155
Email: NSWPharmacyTrial@newcastle.edu.au

Regarding Research Project: **NSW Government-Sponsored Clinical Trial: Management of Urinary Tract Infections by Community Pharmacists (Intervention Study) – Information for Pharmacists – NSW**

Dear Pharmacist,

You are invited to participate in the research project which is being conducted by a consortium of researchers led by the University of Newcastle, The George Institute for Global Health (TGI) and the Hunter Medical Research Institute. Please note that the pharmacy you will deliver the UTI service from will also need to be approved and would need to voluntarily provide consent for their participation. A separate form is available for pharmacy involvement. This project has been funded by the NSW Government.

The information below provides details about the study and how you can participate if you choose to do so.

1. What is the research study about?

The aim of this overall research is to evaluate the implementation, clinical and economic impact of a service model (intervention) delivered by community pharmacists to women aged between 18 years and 65 years in NSW and selected pharmacies in the ACT. You are being invited to participate in a trial of the new service over 10 months.

2. Who is conducting the research?

This research is being conducted under the direction of Dr Sarah Dineen-Griffin from the University of Newcastle's School of Biomedical Sciences and Pharmacy. The evaluation is being led by The George Institute for Global Health in partnership with University of Newcastle and the Hunter Medical Research Institute. The research personnel involved, their affiliations and roles are identified below, under the heading 'Research Team'.

3. Who can participate in the research?

This research study is proactively recruiting pharmacies that meet the criteria outlined in the NSW Health Authority for pharmacies to participate (please see attachment). For your information, we are highlighting the eligibility criteria for an approved pharmacy to participate in the study:

"An 'approved pharmacy' means a pharmacy or class of pharmacies, approved in writing by the Chief Health Officer, which has a service room, consulting room, or area consistent with the following:
o the room or area is not to be used as a dispensary, storeroom, staff room or retail area,
o fully enclosed and provides adequate privacy (a divider or curtain in a dispensary, storeroom, staff room or retail area is not acceptable),
o has adequate lighting,
o is maintained at a comfortable ambient temperature,
o has a hand sanitisation facility,
o has ready access to a hand washing facility, and
o has sufficient floor area, clear of equipment and furniture, to accommodate the person receiving the consultation and an accompanying person, and to allow the pharmacist adequate space to manoeuvre."

Pharmacies must have access to MedAdvisor to complete clinical record keeping for the purposes of the clinical trial assessment.

We are also highlighting the eligibility criteria for an approved pharmacist to participate in the study, which includes that the pharmacist must be:

"employed or engaged in an 'approved pharmacy' who has successfully completed the following training:

- Australasian College of Pharmacy Uncomplicated Cystitis Treatment – Pharmacist Training; or
- Pharmaceutical Society of Australia Managing uncomplicated cystitis; and
- A series of study training module(s) that have been approved by the Chief Health Officer for the purposes of the clinical trial."

A pharmacist is eligible to participate if they hold general registration as a pharmacist with the Australian Health Practitioner Regulation Agency (AHPRA). Pharmacists with provisional registration (intern pharmacists) and pharmacists with conditions on their registration are not eligible to participate in the trial.

If you (or the pharmacy you work in) does not meet these eligibility criteria, your pharmacy will not be able to participate in the study.

If you do not operate within the requirements of the NSW Health Authority or the clinical management protocol, the coverage of your indemnity insurance may be impacted.

4. What does participation involve?

If you agree to participate, you will be asked to complete an online training program (as identified above), if you have not already completed one, which may take up to 6 hours depending on your progress.

You will be asked to deliver a UTI service (approximately 10 minutes per patient consult) to female patients 18 years of age or over and up to and including aged 65 years presenting to your community pharmacy with urinary symptoms. You will also be asked to complete training and make a clinical record of the patient consultation according to an agreed protocol over approximately 10 months. You will be required to screen participants for eligibility to receive the service, provide study information and obtain informed consent from patients. The training programs can be completed at any time. If you wish to complete the training during working hours, there will need to be a private arrangement between you and your employer. In addition to providing the service to the patient, the researchers will visit/contact you to discuss the implementation process in your pharmacy throughout the trial period. The research team anticipate at least monthly touchpoints per pharmacy for support during the trial period, however in some instances, this may be more than monthly. If your pharmacy is deemed to need more support, you will be provided with additional resources, as required. Support may involve a member of the research team visiting or calling the pharmacy, completing checklists to capture barriers and facilitators to service delivery and implementation, and may provide the pharmacy with strategies and solutions to overcome the identified practice barriers. This support may be provided face to face, by phone or by Zoom.

You may also be contacted by the research team with an invitation to participate in a virtual or face-to-face individual interview during or outside business hours. The interview will be recorded and will take up to 60 minutes. You will be asked a series of questions with the aim of gaining a better understanding of the acceptability and effectiveness of the community pharmacy service for women with symptoms of urinary tract infection (UTI).

5. Do you have to take part in this research study?

No. Participation in this research is voluntary. If you do not want to take part, you do not have to. And, if you do agree to participate, you have the option of withdrawing at any time, and we will thank you for your time.

If you do not wish to participate, your decision regarding participation should not impact on your relationship with your employer.

It is not necessary for all pharmacists that work in the pharmacy to provide the service.

6. What is the benefit of participating in this research study?

We cannot guarantee or promise that you will receive any benefits from this research; however, findings of this study may be of potential benefit to you in the future. This may include providing evidence to the NSW

Government on the value of pharmacies and pharmacists as part of the national health reform agenda and expanding the scope of practice for community pharmacy.

A payment of $20 per patient consultation will be made to the pharmacy, irrespective of the consult outcome. Data on these consultations will be made available to the research team, through MedAdvisor software, or any other approved system by the NSW Ministry of Health.

If you are asked to participate in an interview, to minimise inconvenience, you will be i) invited to complete the interview at a time best suited to you; ii) you will be offered a voucher of $50 for their time to participate.

7. Are there any risks associated with your participation in this research?

There are minimal risks associated with this research; however unexpected risks may occur. Inconvenience may occur due to the time taken to complete consultations with patients, and training. Pharmaceutical Defence Limited (PDL) will cover pharmacists as part of their normal indemnity for delivery of this service, following notification of participation.

If you are asked to participate in an interview, it is not expected that interview topics will cause any harm or distress. During interviews if you do experience discomfort from the topics discussed, you can ask to stop or pause the interview or skip any questions.

You can also contact the Chief Investigator, Dr Sarah Dineen-Griffin on 02 4055 0155 or NSWPharmacyTrial@newcastle.edu.au, should you need support regarding any of the issues raised in the research activities.

8. How will your privacy be protected?

Any information obtained in connection with this study that can identify you and the pharmacy in which you provide the service will remain confidential. An ID code will be assigned to you through MedAdvisor or any other approved program by the Chief Medical Officer of NSW Health. Data will be retained securely for a minimum period of fifteen years from the completion of the research project. Information will be managed and stored in accordance with the University's Research Data and Materials Management Guideline or any successor Guideline as well as any other applicable University of Newcastle and TGI policy provisions.

9. How will information collected by the research team be used?

Findings will be reported to the NSW Government, in peer-reviewed publications and conference presentations. Individual participants will not be identifiable in any of the publications generated. Non-identifiable data may be shared with other parties as part of a peer-review process to verify the robustness and integrity of the study, or to contribute to further research and public knowledge. If you would like a copy of the summary of the results, please provide your email address, or mailing address, on the consent form so a summary of the results can be shared with you. The data generated will be used as part of a PhD thesis by Simone Diamandis, Mitchell Budden and Victoria Chisari (under the supervision of Dr Sarah Dineen-Griffin).

10. What you need to do to provide consent to participate?

Read this Information Statement in its entirety and be sure you understand all of the information provided before agreeing to participate. If there is anything you do not understand, or if you have questions, contact the Chief Investigator, Dr Sarah Dineen-Griffin on 02 4055 0155 or NSWPharmacyTrial@newcastle.edu.au for more information. If you decide for your pharmacy to participate, please complete and sign the Consent Form via the Docusign link which will be shared with you by the research team. If you do not respond within one week of the initial invitation, a second invitation will be sent. If you do not respond within 3 working days of the second invitation, we will presume that you do not want to participate. We will not contact you any further after this time.

11. What if you want to withdraw from the research study?

You may withdraw from the study at any time, without providing a reason, by contacting the Chief Investigator, Dr Sarah Dineen-Griffin on 02 4055 0155 or NSWPharmacyTrial@newcastle.edu.au. Once

collected, data cannot be withdrawn from the study. Your decision not to participate or to withdraw from the study will not affect your relationship with the University of Newcastle or any of the other organisations involved in this research.

12. Do you need more information?

If you would like more information about this research project and your potential involvement, please contact the Chief Investigator, Dr Sarah Dineen-Griffin on 02 4055 0155 or NSWPharmacyTrial@newcastle.edu.au.

13. Research Team

- Dr Sarah Dineen-Griffin, The University of Newcastle (Chief Investigator)
- Emeritus Professor Shalom I Benrimoj, The University of Sydney (Co-Investigator)
- Professor David Peiris, The George Institute for Global Health (Co-Investigator)
- Dr Belinda Ford, The George Institute for Global Health (Co-Investigator)
- Dr Gill Schierhout, The George Institute for Global Health (Co-Investigator)
- Dr Anna Campain, The George Institute for Global Health (Co-Investigator)
- Associate Professor Kris Rogers, University of Technology Sydney (Co-Investigator)
- Associate Professor Penny Reeves, Hunter Medical Research Institute (Co-Investigator)
- Emeritus Professor Julie Byles AO, Hunter Medical Research Institute (Co-Investigator)
- Dr Indy Sandaradura, Centre for Infectious Diseases and Microbiology (Co-Investigator)
- Dr Leanne Holt, Macquarie University (Co-Investigator)
- Dr Kylie Gwynne, Macquarie University (Co-Investigator)
- Professor Kylie Williams, University of Technology Sydney (Co-Investigator)
- Dr Helen Benson, University of Technology Sydney (Co-Investigator)
- Associate Professor John Rae, Charles Sturt University (Co-Investigator)
- Ms Anna Barwick, University of New England (Co-Investigator)
- Dr Joanna Moullin, Curtin University (Co-Investigator)
- Ms Jan Donovan, Consumer representative (Co-Investigator)
- Simone Diamandis, The University of Newcastle (PhD Student) – the data will contribute to her PhD thesis.
- Mitchell Budden, The University of Newcastle (PhD Student) – the data will contribute to his PhD thesis.
- Victoria Chisari, The University of Technology Sydney (PhD Student) – the data will contribute to her PhD thesis.

Thank you for your time and consideration of this information.

Dr Sarah Dineen-Griffin
Chief Investigator
University of Newcastle

Concerns or complaints about this research

This project has been approved by the University's Human Research Ethics Committee, Approval No. H-[2023-0119]. If you have concerns about your rights as a participant in this research, or if you have a complaint about the manner in which the research is conducted, please contact the Chief Investigator, Dr Sarah Dineen-Griffin on 02 4055 0155 or NSWPharmacyTrial@newcastle.edu.au. If you would prefer to contact someone independent of the research project, you can forward your concerns to:

Human Research Ethics Officer
Research and Innovation Services
University of Newcastle
University Drive
Callaghan NSW 2308, Australia
Phone: (02) 4921 6333
Email: Human-Ethics@newcastle.edu.au

Human Research Ethics

Research Participant Consent Form (Pharmacist - NSW)

Research Project Information

NSW Government-Sponsored Clinical Trial: Management of Urinary Tract Infections by Community Pharmacists (Intervention Study) – Information for Pharmacist – NSW

Dr Sarah Dineen-Griffin
College of Health, Medicine and Wellbeing
University of Newcastle
Phone number: 02 4055 0155
Email: NSWPharmacyTrial@newcastle.edu.au

Participant Consent

I agree to participate in the above research project and give my consent freely.

I confirm that I hold general registration as a pharmacist with the Australian Health Practitioner Regulation Agency (AHPRA). I confirm that I do not have any conditions on my registration as a pharmacist.

I confirm that I meet all the criteria for the study requirements and the requirements of the NSW Health Authority.

I confirm that I have read and understood the NSW Health Authority to supply a specified restricted substance in relation to the trial and conditions which outline supply.

I have confirmed with my insurance provider that I have professional indemnity insurance to cover the delivery of the UTI service according to the agreed protocol and NSW Health Authority.

I confirm that I have completed the approved UTI training indicated in the NSW Health Authority.

I confirm that I have completed the additional University of Newcastle NSW trial study modules.

I understand that the project will be conducted as described in the Participant Information Statement, a copy of which I have read and retained.

I understand that can withdraw from the project as explained in the Participant Information Statement, and I do not have to give any reason for my decision to withdraw.

I consent to participating in a 10 month study which may involve me delivering a new service to patients in an approved pharmacy and documenting data.

I understand that my personal information will remain confidential to the researchers, except as required by law.

I consent for MedAdvisor software, or an approved system by the Ministry of Health, to provide patient consultation and my data to the research team.

I consent to the research team informing Pharmaceutical Defence Limited, or any other insurer, of my participation in the study.

I understand my pharmacy will be visited/contacted by the research team at least once throughout the main study to discuss implementation of the service in your pharmacy.

I have had the opportunity to have questions answered to my satisfaction.

Human Research Ethics

Research Participant Consent Form (Pharmacist - NSW)

THE UNIVERSITY OF NEWCASTLE AUSTRALIA

Participant Details

Pharmacy name (please print)	
Pharmacist name (please print)	
Signature	
Email address	
Phone number	
Date	

Receiving Research Results

I would like the research team to send me a summary of the research results once available.

☐ Yes

☐ No

If yes, I would like a copy sent to me via:

☐ the email address noted on this form

☐ another address (please specify): *[insert address]*

Human Research Ethics

Participant Information Statement (Pharmacist - ACT)

Clinical Research

Dr Sarah Dineen-Griffin
College of Health, Medicine and Wellbeing
University of Newcastle
Phone number: 02 4055 0155
Email: NSWPharmacyTrial@newcastle.edu.au

Regarding Research Project: **NSW Government-Sponsored Clinical Trial: Management of Urinary Tract Infections by Community Pharmacists (Intervention Study) – Information for Pharmacists – ACT**

Dear Pharmacist,

You are invited to participate in the research project which is being conducted by a consortium of researchers led by the University of Newcastle, The George Institute for Global Health (TGI) and the Hunter Medical Research Institute. Please note that the pharmacy you will deliver the UTI service from will also need to be approved and would need to voluntarily provide consent for their participation. A separate form is available for pharmacy involvement. This project has been funded by the NSW Government.

The information below provides details about the study and how you can participate if you choose to do so.

1. What is the research study about?

The aim of this overall research is to evaluate the implementation, clinical and economic impact of a service model (intervention) delivered by community pharmacists to women aged between 18 years and 65 years in NSW and selected pharmacies in the ACT. You are being invited to participate in a trial of the new service over 10 months.

2. Who is conducting the research?

This research is being conducted under the direction of Dr Sarah Dineen-Griffin from the University of Newcastle's School of Biomedical Sciences and Pharmacy. The research personnel involved, their affiliations and roles are identified below, under the heading 'Research Team'.

3. Who can participate in the research?

Pharmacies in the ACT will be granted a licence by ACT Health to participate in the trial and provide the UTI service (please find attached a PDF copy of the licence application). For your information, we are highlighting the eligibility criteria for an approved pharmacy to participate in the study:

"An 'approved pharmacy' means a pharmacy or class of pharmacies, approved in writing by the Chief Health Officer, which has a service room, consulting room, or area consistent with the following:
o the room or area is not to be used as a dispensary, storeroom, staff room or retail area,
o fully enclosed and provides adequate privacy (a divider or curtain in a dispensary, storeroom, staff room or retail area is not acceptable),
o has adequate lighting,
o is maintained at a comfortable ambient temperature,
o has a hand sanitisation facility,
o has ready access to a hand washing facility, and
o has sufficient floor area, clear of equipment and furniture, to accommodate the person receiving the consultation and an accompanying person, and to allow the pharmacist adequate space to manoeuvre."

Pharmacies must have access to MedAdvisor to complete clinical record keeping for the purposes of the clinical trial assessment.

We are also highlighting the eligibility criteria for an approved pharmacist to participate in the study, which includes that the pharmacist must be:

"employed or engaged in an 'approved pharmacy' who has successfully completed the following training:

- Australasian College of Pharmacy Uncomplicated Cystitis Treatment – Pharmacist Training; or
- Pharmaceutical Society of Australia Managing uncomplicated cystitis; and
- A series of study training module(s) that have been approved by the Chief Health Officer for the purposes of the clinical trial."

A pharmacist is eligible to participate if they hold general registration as a pharmacist with the Australian Health Practitioner Regulation Agency (AHPRA). Pharmacists with provisional registration (intern pharmacists) and pharmacists with conditions on their registration are not eligible to participate in the trial.

If you (or the pharmacy you work in) does not meet these eligibility criteria, your pharmacy will not be able to participate in the study.

If you do not operate within these requirements or the clinical management protocol, the coverage of your indemnity insurance may be impacted.

4. What does participation involve?

If you agree to participate, you will be asked to complete an online training program (as identified above), if you have not already completed one, which may take up to 6 hours depending on your progress.

You will be asked to deliver a UTI service (approximately 10 minutes per patient consult) to female patients 18 years of age or over and up to and including aged 65 years presenting to your community pharmacy with urinary symptoms. You will also be asked to complete a training program and make a clinical record of the patient consultation according to an agreed protocol over approximately 10 months. You will be required to screen participants for eligibility to receive the service, provide study information and obtain informed consent from patients. The training programs can be completed at any time. If you wish to complete the training during working hours, there will need to be a private arrangement between you and your employer. In addition to providing the service to the patient, the researchers will visit/contact you to discuss the implementation process in your pharmacy throughout the trial period. The research team anticipate at least monthly touchpoints per pharmacy for support during the trial period, however in some instances, this may be more than monthly. If your pharmacy is deemed to need more support, you will be provided with additional resources, as required. Support may involve a member of the research team visiting or calling the pharmacy, completing checklists to capture barriers and facilitators to service delivery and implementation, and may provide the pharmacy with strategies and solutions to overcome the identified practice barriers. This support may be provided face to face, by phone or by Zoom.

You may also be contacted by the research team with an invitation to participate in a virtual or face-to-face individual interview during or outside business hours. The interview will be recorded and will take up to 60 minutes. You will be asked a series of questions with the aim of gaining a better understanding of the acceptability and effectiveness of the community pharmacy service for women with symptoms of urinary tract infection (UTI).

5. Do you have to take part in this research study?

No. Participation in this research is voluntary. If you do not want to take part, you do not have to. And, if you do agree to participate, you have the option of withdrawing at any time, and we will thank you for your time.

If you do not wish to participate, your decision regarding participation should not impact on your relationship with your employer.

It is not necessary for all pharmacists that work in the pharmacy to provide the service.

6. What is the benefit of participating in this research study?

We cannot guarantee or promise that you will receive any benefits from this research; however, findings of this study may be of potential benefit to you in the future. This may include providing evidence to the NSW Government on the value of pharmacies and pharmacists as part of the national health reform agenda and expanding the scope of practice for community pharmacy.

A consultation fee will be paid for by the patient to the pharmacy, irrespective of the consult outcome. Data on these consultations will be made available to the research team, through MedAdvisor software, or any other approved system by the NSW Ministry of Health.

If you are asked to participate in an interview, to minimise inconvenience, you will be i) invited to complete the interview at a time best suited to you; ii) you will be offered a voucher of $50 for their time to participate.

7. Are there any risks associated with your participation in this research?

There are minimal risks associated with this research; however unexpected risks may occur. Inconvenience may occur due to the time taken to complete consultations with patients, and training. Pharmaceutical Defence Limited (PDL) will cover pharmacists as part of their normal indemnity for delivery of this service following notification of participation.

If you are asked to participate in an interview, it is not expected that interview topics will cause any harm or distress. During interviews if you do experience discomfort from the topics discussed, you can ask to stop or pause the interview or skip any questions.

You can also contact the Chief Investigator, Dr Sarah Dineen-Griffin on 02 4055 0155 or NSWPharmacyTrial@newcastle.edu.au, should you need support regarding any of the issues raised in the research activities.

8. How will your privacy be protected?

Any information obtained in connection with this study that can identify you and the pharmacy in which you provide the service will remain confidential. An ID code will be assigned to you through MedAdvisor or any other approved program by the Chief Medical Officer of NSW Health. Data will be retained securely for a minimum period of fifteen years from the completion of the research project. Information will be managed and stored in accordance with the University's Research Data and Materials Management Guideline or any successor Guideline as well as any other applicable University of Newcastle and TGI policy provisions.

9. How will information collected by the research team be used?

Findings will be reported to the NSW Government, in peer-reviewed publications and conference presentations. Individual participants will not be identifiable in any of the publications generated. Non-identifiable data may be shared with other parties as part of a peer-review process to verify the robustness and integrity of the study, or to contribute to further research and public knowledge. If you would like a copy of the summary of the results, please provide your email address, or mailing address, on the consent form so a summary of the results can be shared with you. The data generated will be used as part of a PhD thesis by Simone Diamandis, Mitchell Budden and Victoria Chisari (under the supervision of Dr Sarah Dineen-Griffin).

10. What you need to do to provide consent to participate?

Read this Information Statement in its entirety and be sure you understand all of the information provided before agreeing to participate. If there is anything you do not understand, or if you have questions, contact the Chief Investigator, Dr Sarah Dineen-Griffin on 02 4055 0155 or NSWPharmacyTrial@newcastle.edu.au for more information. If you decide for your pharmacy to participate, please complete and sign the Consent Form via the DocuSign link which will be shared with you by the research team. If you do not respond within one week of the initial invitation, a second invitation will be sent. If you do not respond within 3 working days of the second invitation, we will presume that you do not want to participate. We will not contact you any further after this time.

11. What if you want to withdraw from the research study?

You may withdraw from the study at any time, without providing a reason, by contacting the Chief Investigator, Dr Sarah Dineen-Griffin on 02 4055 0155 or NSWPharmacyTrial@newcastle.edu.au. Once collected, data cannot be withdrawn from the study. Your decision not to participate or to withdraw from the study will not affect your relationship with the University of Newcastle or any of the other organisations involved in this research.

12. Do you need more information?

If you would like more information about this research project and your potential involvement, please contact the Chief Investigator, Dr Sarah Dineen-Griffin on 02 4055 0155 or NSWPharmacyTrial@newcastle.edu.au.

13. Research Team

- Dr Sarah Dineen-Griffin, The University of Newcastle (Chief Investigator)
- Emeritus Professor Shalom I Benrimoj, The University of Sydney (Co-Investigator)
- Professor David Peiris, The George Institute for Global Health (Co-Investigator)
- Dr Belinda Ford, The George Institute for Global Health (Co-Investigator)
- Dr Gill Schierhout, The George Institute for Global Health (Co-Investigator)
- Dr Anna Campain, The George Institute for Global Health (Co-Investigator)
- Associate Professor Kris Rogers, University of Technology Sydney (Co-Investigator)
- Associate Professor Penny Reeves, Hunter Medical Research Institute (Co-Investigator)
- Emeritus Professor Julie Byles AO, Hunter Medical Research Institute (Co-Investigator)
- Dr Indy Sandaradura, Centre for Infectious Diseases and Microbiology (Co-Investigator)
- Dr Leanne Holt, Macquarie University (Co-Investigator)
- Dr Kylie Gwynne, Macquarie University (Co-Investigator)
- Professor Kylie Williams, University of Technology Sydney (Co-Investigator)
- Dr Helen Benson, University of Technology Sydney (Co-Investigator)
- Associate Professor John Rae, Charles Sturt University (Co-Investigator)
- Ms Anna Barwick, University of New England (Co-Investigator)
- Dr Joanna Moullin, Curtin University (Co-Investigator)
- Ms Jan Donovan, Consumer representative (Co-Investigator)
- Simone Diamandis, The University of Newcastle (PhD Student) – the data will contribute to her PhD thesis.
- Mitchell Budden, The University of Newcastle (PhD Student) – the data will contribute to his PhD thesis.
- Victoria Chisari, The University of Technology Sydney (PhD Student) – the data will contribute to her PhD thesis.

Thank you for your time and consideration of this information.

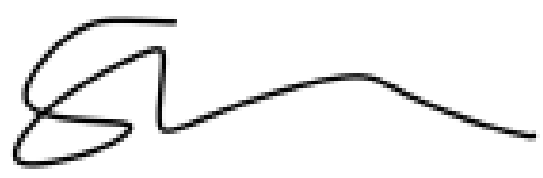

Dr Sarah Dineen-Griffin
Chief Investigator
University of Newcastle

Concerns or complaints about this research

This project has been approved by the University's Human Research Ethics Committee, Approval No. H-[2023-0119]. If you have concerns about your rights as a participant in this research, or if you have a complaint about the manner in which the research is conducted, please contact the Chief Investigator, Dr Sarah Dineen-Griffin on 02 4055 0155 or NSWPharmacyTrial@newcastle.edu.au. If you would prefer to contact someone independent of the research project, you can forward your concerns to:

Human Research Ethics Officer
Research and Innovation Services
University of Newcastle
University Drive
Callaghan NSW 2308, Australia
Phone: (02) 4921 6333
Email: Human-Ethics@newcastle.edu.au

Human Research Ethics

Research Participant Consent Form (Pharmacist - ACT)

THE UNIVERSITY OF NEWCASTLE AUSTRALIA

Research Project Information

NSW Government-Sponsored Clinical Trial: Management of Urinary Tract Infections by Community Pharmacists (Intervention Study) – Information for Pharmacist – ACT

Dr Sarah Dineen-Griffin
College of Health, Medicine and Wellbeing
University of Newcastle
Phone number: 02 4055 0155
Email: NSWPharmacyTrial@newcastle.edu.au

Participant Consent

I agree to participate in the above research project and give my consent freely.

I confirm that I hold general registration as a pharmacist with the Australian Health Practitioner Regulation Agency (AHPRA). I confirm that I do not have any conditions on my registration as a pharmacist.

I confirm that I meet all the criteria for the study requirements and the requirements of the ACT Health Licence.

I confirm that I have read and understood the NSW Health Authority to supply a specified restricted substance in relation to the trial and conditions which outline supply.

I have confirmed with my insurance provider that I have professional indemnity insurance to cover the delivery of the UTI service according to the agreed protocol and NSW Health Authority.

I confirm that I have completed the approved UTI training indicated in the NSW Health Authority.

I confirm that I have completed the additional University of Newcastle NSW trial study modules.

I understand that the project will be conducted as described in the Participant Information Statement, a copy of which I have read and retained.

I understand that can withdraw from the project as explained in the Participant Information Statement, and I do not have to give any reason for my decision to withdraw.

I consent to participating in a 10 month study which may involve me delivering a new service to patients in an approved pharmacy and documenting data.

I understand that my personal information will remain confidential to the researchers, except as required by law.

I consent for MedAdvisor software, or an approved system by the Ministry of Health, to provide patient consultation and my data to the research team.

I consent to the research team informing Pharmaceutical Defence Limited, or any other insurer, of my participation in the study.

I understand my pharmacy will be visited/contacted by the research team at least once throughout the feasibility study to discuss implementation of the service in your pharmacy.

I have had the opportunity to have questions answered to my satisfaction.

Human Research Ethics

Research Participant Consent Form (Pharmacist - ACT)

Participant Details

Pharmacy name (please print)	
Pharmacist name (please print)	
Signature	
Email address	
Phone number	
Date	

Receiving Research Results

I would like the research team to send me a summary of the research results once available.

☐ Yes

☐ No

If yes, I would like a copy sent to me via:

☐ the email address noted on this form

☐ another address (please specify): *[insert address]*

Human Research Ethics

Participant Information Statement (Patient - NSW)

Clinical Research

Dr Sarah Dineen-Griffin
College of Health, Medicine and Wellbeing
University of Newcastle
Phone number: 02 4055 0155
Email: NSWPharmacyTrial@newcastle.edu.au

Regarding Research Project: **NSW Government-Sponsored Clinical Trial: Management of Urinary Tract Infections by Community Pharmacists (Intervention Study) – Information for Patients - NSW**

You are invited to participate in the research project noted above which is being conducted by a consortium of researchers led by the University of Newcastle, The George Institute for Global Health (TGI) and the Hunter Medical Research Institute. This project has been funded by the NSW Government. The information below provides more detail about the study and how you can participate if you choose to do so.

1. What is the research study about?

The aim of this overall research is to evaluate the implementation, clinical and economic impact of a service model (intervention) delivered by community pharmacists to women aged between 18 years and 65 years in NSW and selected pharmacies in the ACT.

2. Who is conducting the research?

This research is being conducted under the direction of Dr Sarah Dineen-Griffin from the University of Newcastle's School of Biomedical Sciences and Pharmacy. The evaluation is being led by The George Institute for Global Health in partnership with University of Newcastle and the Hunter Medical Research Institute. The personnel involved, their affiliations and roles are identified below, under the heading 'Research Team' below.

3. Who can participate in the research?

Eligible patients will be females aged between 18 years and 65 years presenting to community pharmacies in NSW, and select pharmacies in the ACT, with symptoms associated with an uncomplicated urinary tract infection. Due to potential complications, this service is only available to patients that are anatomically female at birth.

You are invited to participate and will be asked to provide consent if you agree to participate.

4. What does participation involve?

You are being asked to participate because the pharmacy you are attending today is participating in a research trial. We will ask you to participate in:

- An initial eligibility assessment.
- 10-minute consultation with the pharmacist. In this consultation your pharmacist will ask and record your symptoms, medical conditions, and other clinical information they would require ensuring an appropriate clinical decision is made on the management of your symptoms. For example, the pharmacist will ask you about your personal medical history, the medications you are taking, and details about the symptoms you are experiencing to ensure that the care and advice delivered to you is appropriate. You will receive care from your pharmacist based on your symptoms. This may include educating you on how to self-manage your condition and supply you a medicine, if appropriate.
- 5 minute follow up telephone or text message with a link to a questionnaire one week after your visit to the pharmacy with a researcher.
- A record of the treatment supplied will be shared with your usual general practitioner, and this may also include your personal or health information.
- A record of the consultation will be shared with the researchers.

There will be no charge to you for the consultation with the pharmacist, however there will be a cost to you if a medicine or any other product is supplied.

We are also seeking your permission to access information on use of hospital services (emergency department, hospital admission, and specialist appointments) to help us understand if you used any other health care during the trial. There is additional information and a consent form to complete for this aspect of the project. The release of your Services Australia personal information to the study is completely voluntary and there will be no cost to you. If you do not want to consent to the release of your information you do not have to. You should feel under no obligation to consent to the Study. Choosing not to consent to the release of your information will not affect your current and future medical care in any way. See the separate MBS/PBS Participant Information and Consent Form.

A small number of participants will also be invited to participate in a follow-up individual interview lasting around 30 minutes conducted virtually at a time that is convenient for you. You will be asked a series of questions on your experiences of health care and awareness and perceptions of services available. Interviews will be digitally recorded and transcribed using a professional transcription service. As part of the consent process, participants will be asked if they would like to receive a copy of their interview transcript.

5. Do you have to take part in this research study?

No. Participation in this research study is voluntary. If you do not want to take part, you do not have to. If you do not wish to participate, the pharmacist will provide you with your usual care, or advise you to seek alternative health advice. And, if you do agree to participate, you have the option of withdrawing at any time, and will thank you for your time. Your decision to withdraw will not affect the health care you receive.

6. What is the benefit of participating in this research study?

We cannot guarantee or promise that you will receive any benefits from this research; however, findings of this study may be of potential benefit to patients, like you, in the future. This may include providing evidence to the NSW Government on the value of pharmacies and pharmacists as part of the national health reform agenda and expanding the scope of practice for community pharmacy.

If you are asked to participate in an interview, to minimise inconvenience, you will be i) invited to complete the group interview at a time best suited to you; ii) you will be offered a voucher of $20 in recognition of the time taken out of your usual day to participate.

7. Are there any risks associated with your participation in this research?

There are minimal risks associated with this research; however unexpected risks may occur. If you experience any adverse events (e.g., drug reaction, side effect to a medication, emotional stress, or any unusual symptoms etc.), if your symptoms don't improve or you have any concerns, please contact the pharmacy that provided you the service, or seek alternative health advice. Inconvenience may occur due to the time taken to complete the consultation.

If you are asked to participate in an interview, it is not expected that interview topics will cause any harm or distress. During interviews if you do experience discomfort from the topics discussed or recollection of your health care experiences, you can ask to stop or pause the interview or skip any questions. If you raise any concerns or have questions about symptoms or your health care, you will be directed to discuss this with your usual health care provider.

You can also contact the research team via email at NSWPharmacyTrial@newcastle.edu.au or via phone on 02 4055 0155, should you need support regarding any of the issues raised in the research activities.

8. How will your privacy be protected?

Any information obtained in connection with this study that can identify you will remain confidential. Your information will be anonymized, so individuals won't be exposed in any reports or publications. Data will be retained securely for a minimum period of fifteen years from the completion of the research project. Information will be managed and stored in accordance with the University's Research Data and Materials

Management Guideline or any successor Guideline as well as any other applicable University of Newcastle or TGI policy provisions.

9. How will information collected by the research team be used?

Findings will be reported to the NSW Government, in peer-reviewed publications and conference presentations. Individual participants will not be identifiable in any of the publications generated. Non-identifiable data may be shared with other parties as part of a peer-review process to verify the robustness and integrity of the study, or to contribute to further research and public knowledge. If you would like a copy of the summary of the results, please provide your email address, or mailing address, on the consent form so a summary of the results can be shared with you. The data generated will be used as part of a PhD thesis by Simone Diamandis, Mitchell Budden and Victoria Chisari (under the supervision of Dr Sarah Dineen-Griffin).

10. What you need to do to provide consent to participate

Read this Information Statement in its entirety and be sure you understand all of the information provided before agreeing to participate. If there is anything you do not understand, or if you have questions, you can discuss this with your pharmacist. If you decide to participate, please provide signed consent and the pharmacist will provide you a copy of the Participant Information Sheet and Consent form for your retention via email or printed hard copy, and the pharmacist will proceed to provide you the service.

11. What if you want to withdraw from the research study?

You may withdraw from the study at any time by contacting the Chief Investigator, Dr Sarah Dineen-Griffin on 02 4055 0155 or NSWPharmacyTrial@newcastle.edu.au. Your decision not to participate or to withdraw from the study will not affect your relationship with the University of Newcastle or any of the other organisations involved in this research.

12. Do you need more information?

If you would like more information about this research project and your potential involvement, please contact the Chief Investigator, Dr Sarah Dineen-Griffin on 02 4055 0155 or NSWPharmacyTrial@newcastle.edu.au.

13. Research Team

- Dr Sarah Dineen-Griffin, The University of Newcastle (Chief Investigator)
- Emeritus Professor Shalom I Benrimoj, The University of Sydney (Co-Investigator)
- Professor David Peiris, The George Institute for Global Health (Co-Investigator)
- Dr Belinda Ford, The George Institute for Global Health (Co-Investigator)
- Dr Gill Schierhout, The George Institute for Global Health (Co-Investigator)
- Dr Anna Campain, The George Institute for Global Health (Co-Investigator)
- Associate Professor Kris Rogers, University of Technology Sydney (Co-Investigator)
- Associate Professor Penny Reeves, Hunter Medical Research Institute (Co-Investigator)
- Emeritus Professor Julie Byles AO, Hunter Medical Research Institute (Co-Investigator)
- Dr Indy Sandaradura, Centre for Infectious Diseases and Microbiology (Co-Investigator)
- Dr Leanne Holt, Macquarie University (Co-Investigator)
- Dr Kylie Gwynne, Macquarie University (Co-Investigator)
- Professor Kylie Williams, University of Technology Sydney (Co-Investigator)
- Dr Helen Benson, University of Technology Sydney (Co-Investigator)
- Associate Professor John Rae, Charles Sturt University (Co-Investigator)
- Ms Anna Barwick, University of New England (Co-Investigator)
- Dr Joanna Moullin, Curtin University (Co-Investigator)
- Ms Jan Donovan, Consumer representative (Co-Investigator)
- Simone Diamandis, The University of Newcastle (PhD Student) – the data will contribute to her PhD thesis.
- Mitchell Budden, The University of Newcastle (PhD Student) – the data will contribute to his PhD thesis.

- Victoria Chisari, The University of Technology Sydney (PhD Student) – the data will contribute to her PhD thesis.

Thank you for your time and consideration of this information.

Dr Sarah Dineen-Griffin
Chief Investigator
University of Newcastle

Concerns or complaints about this research

This project has been approved by the University's Human Research Ethics Committee, Approval No. H-[2023-0119]. If you have concerns about your rights as a participant in this research, or if you have a complaint about the manner in which the research is conducted, please contact the Chief Investigator, Dr Sarah Dineen-Griffin on 02 4055 0155 or NSWPharmacyTrial@newcastle.edu.au. If you would prefer to contact someone independent of the research project, you can forward your concerns to:

Human Research Ethics Officer
Research and Innovation Services
University of Newcastle
University Drive
Callaghan NSW 2308, Australia
Phone: (02) 4921 6333
Email: Human-Ethics@newcastle.edu.au

Human Research Ethics

Research Participant Consent Form (Patient - NSW)

Research Project Information

NSW Government-Sponsored Clinical Trial: Management of Urinary Tract Infections by Community Pharmacists (Intervention Study) – Information for Patient - NSW

Dr Sarah Dineen-Griffin
College of Health, Medicine and Wellbeing
University of Newcastle
Phone number: 02 4985 4299
Email: NSWPharmacyTrial@newcastle.edu.au

Participant Consent

I agree to participate in the above research project and give my consent freely.

I understand that the project will be conducted as described in the Participant Information Statement, a copy of which I have read and retained.

I understand that I can withdraw from the project as explained in the Participant Information Statement, and I do not have to give any reason for my decision to withdraw.

I consent to participating in a study which will involve receiving a consultation with a pharmacist about my urinary symptoms.

I consent to a record of the treatment supplied to me being shared with my usual general practitioner, and this may also include my personal or health information.

I consent to provide my name and contact details for a follow up call, text message or email for a short follow up survey on the service I received approximately one week after my visit to the pharmacy.

I understand that I may be invited by the research team to participate in a phone or video interview to find out what I thought about the new service.

I consent to a record of my consultation being shared with the researchers.

I understand there will be no charge for my consultation with the pharmacist, however I will have to pay for any medicines or products supplied.

I consent to the pharmacist accessing my Electronic Health Record, where required and available.

I understand that my personal information will remain confidential to the researchers, except as required by law.

I understand that the research team will access information from NSW Health on use of hospital services.

I understand that the research team will be linking Services Australia data to State health data.

I have had the opportunity to have questions answered to my satisfaction.

Human Research Ethics

Research Participant Consent Form (Patient - NSW)

THE UNIVERSITY OF NEWCASTLE AUSTRALIA

Participant Details

Full name (please print)	
Signature	
Email	
Mobile or landline number	
Date	

Usual GP Details

Doctors full name (please print)	
Medical practice (please print)	

Follow-up phone call/text message by research team at 7 days

You will need to complete a follow-up survey from The George Institute (TGI) in 7 days' time. How would you like to receive this survey?

☐ Text message ☐ Email ☐ Phone call

Interview

I would be happy to be contacted for an interview.

☐ Yes ☐ No

I would like a copy of my interview transcript emailed to me.

☐ Yes ☐ No

Receiving Research Results

I would like the research team to send me a summary of the research results once available.

☐ Yes

☐ No

If yes, I would like a copy sent to me via:

☐ the email address noted on this form

☐ another address (please specify): *[insert address]*

Human Research Ethics
Participant Information Statement (Patient - ACT)
Clinical Research

Dr Sarah Dineen-Griffin
College of Health, Medicine and Wellbeing
University of Newcastle
Phone number: 02 4055 0155
Email: NSWPharmacyTrial@newcastle.edu.au

Regarding Research Project: **NSW Government-Sponsored Clinical Trial: Management of Urinary Tract Infections by Community Pharmacists (Intervention Study) – Information for Patients - ACT**

You are invited to participate in the research project noted above which is being conducted by a consortium of researchers led by the University of Newcastle, The George Institute for Global Health (TGI) and the Hunter Medical Research Institute. This project has been funded by the NSW Government. The information below provides more detail about the study and how you can participate if you choose to do so.

1. What is the research study about?

The aim of this overall research is to evaluate the implementation, clinical and economic impact of a service model (intervention) delivered by community pharmacists to women aged between 18 years and 65 years in NSW and selected pharmacies in the ACT.

2. Who is conducting the research?

This research is being conducted under the direction of Dr Sarah Dineen-Griffin from the University of Newcastle's School of Biomedical Sciences and Pharmacy. The evaluation is being led by The George Institute for Global Health in partnership with University of Newcastle and the Hunter Medical Research Institute. The personnel involved, their affiliations and roles are identified below, under the heading 'Research Team' below.

3. Who can participate in the research?

Eligible patients will be females aged between 18 years and 65 years presenting to community pharmacies in NSW, and select pharmacies in the ACT, with symptoms associated with an uncomplicated urinary tract infection. Due to potential complications, this service is only available to patients that are anatomically female at birth.

You are invited to participate and will be asked to provide consent if you agree to participate.

4. What does participation involve?

You are being asked to participate because the pharmacy you are attending today is participating in a research trial. We will ask you to participate in:

- An initial eligibility assessment.
- 10-minute consultation with the pharmacist. In this consultation your pharmacist will ask and record your symptoms, medical conditions, and other clinical information they would require ensuring an appropriate clinical decision is made on the management of your symptoms. For example, the pharmacist will ask you about your personal medical history, the medications you are taking, and details about the symptoms you are experiencing to ensure that the care and advice delivered to you is appropriate. You will receive care from your pharmacist based on your symptoms. This may include educating you on how to self-manage your condition and supply you a medicine, if appropriate.
- 5 minute follow up telephone or text message with a link to a questionnaire one week after your visit to the pharmacy with a researcher.
- A record of the treatment supplied will be shared with your usual general practitioner, and this may also include your personal or health information.
- A record of the consultation will be shared with the researchers.

There will be a charge to you for the consultation with the pharmacist, and there will be an additional cost to you if a medicine or any other product is supplied.

We are also seeking your permission to access information on use of hospital services (emergency department, hospital admission, and specialist appointments) to help us understand if you used any other health care during the trial. There is additional information and a consent form to complete for this particular aspect of the project. The release of your Services Australia personal information to the study is completely voluntary and there will be no cost to you. If you do not want to consent to the release of your information you do not have to. You should feel under no obligation to consent to the Study. Choosing not to consent to the release of your information will not affect your current and future medical care in any way. See the separate MBS/PBS Participant Information and Consent Form.

A small number of participants will also be invited to participate in a follow-up individual interview lasting around 30 minutes conducted virtually at a time that is convenient for you. You will be asked a series of questions on your experiences of health care and awareness and perceptions of services available. Interviews will be digitally recorded and transcribed using a professional transcription service. As part of the consent process, participants will be asked if they would like to receive a copy of their interview transcript.

5. Do you have to take part in this research study?

No. Participation in this research study is voluntary. If you do not want to take part, you do not have to. If you do not wish to participate, the pharmacist will provide you with your usual care, or advise you to seek alternative health advice. And, if you do agree to participate, you have the option of withdrawing at any time, and will thank you for your time. Your decision to withdraw will not affect the health care you receive.

6. What is the benefit of participating in this research study?

We cannot guarantee or promise that you will receive any benefits from this research; however, findings of this study may be of potential benefit to patients, like you, in the future. This may include providing evidence to the NSW Government on the value of pharmacies and pharmacists as part of the national health reform agenda and expanding the scope of practice for community pharmacy.

If you are asked to participate in an interview, to minimise inconvenience, you will be i) invited to complete the group interview at a time best suited to you; ii) you will be offered a voucher of $20 in recognition of the time taken out of your usual day to participate.

7. Are there any risks associated with your participation in this research?

There are minimal risks associated with this research; however unexpected risks may occur. If you experience any adverse events (e.g., drug reaction, side effect to a medication, emotional stress, or any unusual symptoms etc.), if your symptoms don't improve or you have any concerns, please contact the pharmacy that provided you the service, or seek alternative health advice. Inconvenience may occur due to the time taken to complete the consultation.

If you are asked to participate in an interview, it is not expected that interview topics will cause any harm or distress. During interviews if you do experience discomfort from the topics discussed or recollection of your health care experiences, you can ask to stop or pause the interview or skip any questions. If you raise any concerns or have questions about symptoms or your health care, you will be directed to discuss this with your usual health care provider.

You can also contact the research team via email at NSWPharmacyTrial@newcastle.edu.au or via phone on 02 4055 0155, should you need support regarding any of the issues raised in the research activities.

8. How will your privacy be protected?

Any information obtained in connection with this study that can identify you will remain confidential. Your information will be anonymized, so individuals won't be exposed in any reports or publications. Data will be retained securely for a minimum period of fifteen years from the completion of the research project. Information will be managed and stored in accordance with the University's Research Data and Materials

Management Guideline or any successor Guideline as well as any other applicable University of Newcastle or TGI policy provisions.

9. How will information collected by the research team be used?

Findings will be reported to the NSW Government, in peer-reviewed publications and conference presentations. Individual participants will not be identifiable in any of the publications generated. Non-identifiable data may be shared with other parties as part of a peer-review process to verify the robustness and integrity of the study, or to contribute to further research and public knowledge. If you would like a copy of the summary of the results, please provide your email address, or mailing address, on the consent form so a summary of the results can be shared with you. The data generated will be used as part of a PhD thesis by Simone Diamandis, Mitchell Budden and Victoria Chisari (under the supervision of Dr Sarah Dineen-Griffin).

10. What you need to do to provide consent to participate

Read this Information Statement in its entirety and be sure you understand all of the information provided before agreeing to participate. If there is anything you do not understand, or if you have questions, you can discuss this with your pharmacist. If you decide to participate, please provide signed consent and the pharmacist will provide you a copy of the Participant Information Sheet and Consent form for your retention via email or printed hard copy, and the pharmacist will proceed to provide you the service.

11. What if you want to withdraw from the research study?

You may withdraw from the study at any time by contacting the Chief Investigator, Dr Sarah Dineen-Griffin on 02 4055 0155 or NSWPharmacyTrial@newcastle.edu.au. Your decision not to participate or to withdraw from the study will not affect your relationship with the University of Newcastle or any of the other organisations involved in this research.

12. Do you need more information?

If you would like more information about this research project and your potential involvement, please contact the Chief Investigator, Dr Sarah Dineen-Griffin on 02 4055 0155 or NSWPharmacyTrial@newcastle.edu.au.

13. Research Team

- Dr Sarah Dineen-Griffin, The University of Newcastle (Chief Investigator)
- Emeritus Professor Shalom I Benrimoj, The University of Sydney (Co-Investigator)
- Professor David Peiris, The George Institute for Global Health (Co-Investigator)
- Dr Belinda Ford, The George Institute for Global Health (Co-Investigator)
- Dr Gill Schierhout, The George Institute for Global Health (Co-Investigator)
- Dr Anna Campain, The George Institute for Global Health (Co-Investigator)
- Associate Professor Kris Rogers, University of Technology Sydney (Co-Investigator)
- Associate Professor Penny Reeves, Hunter Medical Research Institute (Co-Investigator)
- Emeritus Professor Julie Byles AO, Hunter Medical Research Institute (Co-Investigator)
- Dr Indy Sandaradura, Centre for Infectious Diseases and Microbiology (Co-Investigator)
- Dr Leanne Holt, Macquarie University (Co-Investigator)
- Dr Kylie Gwynne, Macquarie University (Co-Investigator)
- Professor Kylie Williams, University of Technology Sydney (Co-Investigator)
- Dr Helen Benson, University of Technology Sydney (Co-Investigator)
- Associate Professor John Rae, Charles Sturt University (Co-Investigator)
- Ms Anna Barwick, University of New England (Co-Investigator)
- Dr Joanna Moullin, Curtin University (Co-Investigator)
- Ms Jan Donovan, Consumer representative (Co-Investigator)
- Simone Diamandis, The University of Newcastle (PhD Student) – the data will contribute to her PhD thesis.
- Mitchell Budden, The University of Newcastle (PhD Student) – the data will contribute to his PhD thesis.

- Victoria Chisari, The University of Technology Sydney (PhD Student) – the data will contribute to her PhD thesis.

Thank you for your time and consideration of this information.

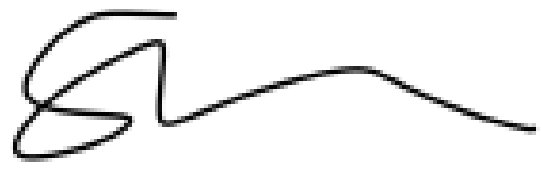

Dr Sarah Dineen-Griffin
Chief Investigator
University of Newcastle

Concerns or complaints about this research

This project has been approved by the University's Human Research Ethics Committee, Approval No. H-[2023-0119]. If you have concerns about your rights as a participant in this research, or if you have a complaint about the manner in which the research is conducted, please contact the Chief Investigator, Dr Sarah Dineen-Griffin on 02 4055 0155 or NSWPharmacyTrial@newcastle.edu.au. If you would prefer to contact someone independent of the research project, you can forward your concerns to:

Human Research Ethics Officer
Research and Innovation Services
University of Newcastle
University Drive
Callaghan NSW 2308, Australia
Phone: (02) 4921 6333
Email: Human-Ethics@newcastle.edu.au

Human Research Ethics

Research Participant Consent Form (Patient - ACT)

Research Project Information

NSW Government-Sponsored Clinical Trial: Management of Urinary Tract Infections by Community Pharmacists (Intervention Study) – Information for Patient - ACT

Dr Sarah Dineen-Griffin
College of Health, Medicine and Wellbeing
University of Newcastle
Phone number: 02 4985 4299
Email: NSWPharmacyTrial@newcastle.edu.au

Participant Consent

I agree to participate in the above research project and give my consent freely.

I understand that the project will be conducted as described in the Participant Information Statement, a copy of which I have read and retained.

I understand that I can withdraw from the project as explained in the Participant Information Statement, and I do not have to give any reason for my decision to withdraw.

I consent to participating in a study which will involve receiving a consultation with a pharmacist about my urinary symptoms.

I consent to a record of the treatment supplied to me being shared with my usual general practitioner, and this may also include my personal or health information.

I consent to provide my name and contact details for a follow up call, text message or email for a short follow up survey on the service I received approximately one week after my visit to the pharmacy.

I understand that I may be invited by the research team to participate in a phone or video interview to find out what I thought about the new service.

I consent to a record of my consultation being shared with the researchers.

I understand there may be a charge for my consultation with the pharmacist, and I will have to pay for any medicines or products supplied.

I consent to the pharmacist accessing my Electronic Health Record, where required and available.

I understand that my personal information will remain confidential to the researchers, except as required by law.

I understand that the research team will access information from NSW Health on use of hospital services.

I understand that the research team will be linking Services Australia data to State health data.

I have had the opportunity to have questions answered to my satisfaction.

Human Research Ethics

Research Participant Consent Form (Patient - ACT)

Participant Details

Full name (please print)	
Signature	
Email	
Mobile or landline number	
Date	

Usual GP Details

Doctors full name (please print)	
Medical practice (please print)	

Follow-up phone call/text message by research team at 7 days

You will need to complete a follow-up survey from The George Institute (TGI) in 7 days' time. How would you like to receive this survey?

☐ Text message ☐ Email ☐ Phone call

Interview

I would be happy to be contacted for an interview.

☐ Yes ☐ No

I would like a copy of my interview transcript emailed to me.

☐ Yes ☐ No

Receiving Research Results

I would like the research team to send me a summary of the research results once available.

☐ Yes

☐ No

If yes, I would like a copy sent to me via:

☐ the email address noted on this form

☐ another address (please specify): *[insert address]*

NSW Government-Sponsored Clinical Trial: Management of Urinary Tract Infections by Community Pharmacists (Intervention Study) - MBS/PBS Participant Information and Consent Form

Important information

Services Australia is not involved in the conduct of this study other than to release your Medicare Benefits Schedule (MBS) and/or Pharmaceutical Benefits Scheme (PBS) claims information. Services Australia will not provide your personal information to the Management of UTI by Community Pharmacists Study (the Study) without your consent. To agree to the release of your information you must complete the 'Services Australia Participant Consent Form'.

You will be asked to sign a consent form authorising the study to access your complete MBS and/or PBS as outlined in the consent form. Medicare collects information on your doctor visits and the associated costs, while the PBS collects information on the prescription medications you have filled at pharmacies.

The release of your Services Australia personal information to the Study is completely voluntary and there will be no cost to you. If you do not want to consent to the release of your information you do not have to. You should feel under no obligation to consent to the Study. Choosing not to consent to the release of your information will not affect your current and future medical care in any way.

What is the Study about?

This study is a 10 month study to assess whether a model in which community pharmacists will be able to prescribe antibiotics for uncomplicated UTIs is safe, effective and acceptable to women aged 18-65 years. The Study will assess whether the treatments provided by pharmacists result in resolution of symptoms. It will also assess whether appropriate referrals are made to GPs and other care providers if needed and whether women find the service satisfactory.

Your consent

If you agree to us obtaining this data, you will be asked to give your consent to indicate that you have read and understood this information, and that you freely agree to the conditions described in this participant information sheet. Please print or save the Participant Information Sheet and Consent Form for your records.

Who runs the Study?

The chief investigator for the overall study is Dr Sarah Dineen-Griffin, Lecturer (Pharmacy), The University of Newcastle. The chief investigator for the Phase 3 intervention evaluation study is Professor David Peiris, Chief Scientist, The George Institute for Global Health, UNSW Sydney.

Does the study have approval from an ethics committee?

The study has been approved by the Human Research Ethics Committee of University of Newcastle. This study will be carried out according to the National Statement on Ethical Conduct in Human Research (2007, updated 2018) (Approval No. H-[2023-0119]).

Services Australia has confirmed that a Human Research Ethics Committee (HREC) that is registered with the National Health and Medical Research Council (NHMRC) and operates within guidelines set out by the NHMRC has approved this research and any associated documents.

Do I have to participate in this component of the study?

Your participation in this component of the study is completely voluntary and there will be no cost to you. If you do not want to take part in this study, you do not have to. You should feel under no obligation to participate in this study. Choosing not to take part in this study will not affect your current and future medical care in any way.

What if I no longer want to participate?

You are under no obligation to continue with the consented release of your Services Australia information. You may change your mind at any time about releasing your information to the Study. People withdraw from studies for various reasons, and you do not need to provide a reason.

You can withdraw your consent to release your Services Australia information by completing and signing the 'Services Australia Participant Withdrawal of Consent Form'. This form is to be completed by you and supplied to the research team if you choose to withdraw your consent at a later date. If you withdraw your consent to release your information to the study, you will be able to choose whether the study will destroy or retain your Services Australia information it has collected about you. You should only choose one of these options. Where both boxes are ticked in error or neither box is ticked, the study will destroy all information it has collected about you. If you do withdraw your consent from the study and your information has already been analysed and/or included in a publication, your personal information may not be able to be withdrawn or destroyed. In such circumstances, your personal information will continue to form part of the project study records and results. Your privacy will continue to be protected at all times.

What does the study involve?

For this component of the study, we are asking for consent to:

i. link with records held by the Pharmaceutical Benefits Scheme (PBS) to investigate the use of medicines associated with your health care in the period 12 months before and 12 months after your visit to the pharmacy. This will provide information on use of antibiotics and other medicines.
ii. link with records held by the Medicare Benefits Schedule (MBS) on diagnostic procedures and investigations, imaging services, pathology services and therapeutic procedures in the 12 months before and 12 months after your visit to the pharmacy. This will enable us to obtain a comprehensive picture of what health services you have used before and after enrolling in the study.

The information obtained will be stored with identifiers removed and will only be available for use by the Study team. This information will be retained for at least 15 years. At the end of this time either a request will be made to retain this data for an additional period, or the electronic data will be destroyed (including archive versions but excluding where data has been incorporated into analysis files used for publication of findings).

Your personal information specified within the consent form will be sent securely to Services Australia to authorise the release of your Services Australia information to the Study. Services Australia will retain your consent form for the life of the study as a record of your consent. A copy of your consent form will also be retained by the Study for the life of the study. Your Services Australia information will be de-identified and stored securely by the Study on servers, or hosted through cloud computing providers, physically located within Australian borders. Your Services Australia information will not be sent outside of Australian jurisdiction and is governed by the Privacy Act 1988.

Your Services Australia information that has been included in de-identified databases will be securely destroyed after the final publication of the study. However, if you withdraw from the Study you can request the destruction of your Services Australia information, provided it has not been deidentified, analysed and published. All information will be securely destroyed at the completion of the study in a manner appropriate to the security classification of the record content.

How are my privacy and confidentiality protected?

All electronic records containing personal information held by the George Institute for Global Health are accessed via password protected servers in an ISO 27001 certified data centre in Sydney. Data are stored in compliance with the Commonwealth Privacy Act.

The George Institute for Global Health is subject to privacy and health records laws which regulate how it collects and handles personal information, including sensitive and health information. Further details about how it handles your personal information is set out in its Privacy Policy (as amended from time to time), available on George Institute's website https://www.georgeinstitute.org.au/privacy-policy. You should review our Privacy Policy regularly, as it may be amended during the course of the Study to reflect changes to our practices and legal obligations.

Access to information that identifies you, such as your name, date of birth and address, will be restricted to staff employed by the George Institute to maintain contact with you for the duration of your participation in this study.

Government agencies and publishers of scientific journals sometimes require research data to be available via open or mediated access data repositories that meet international security and safety standards for sharing data. In such cases, no individually identifiable data would be provided. We will not include your name or other identifying information in any publication.

In accordance with relevant Australian and/or New South Wales privacy legislation, you have the right to access the information about you that is collected and held by us. You also have the right to ask for information to be corrected. Contact the study team on 02 8052 4300 or email uti@georgeinstitute.org if you would like to access your information. If you do so, once we verify your identity, you will receive a copy of data obtained at the time of request.

How can I find out what the researchers learn from the study?

All participants will be emailed a summary of the study findings at the end of 10 months which will highlight key findings and future plans for the study. Participation in the study should not alter your use of routine health care or health screenings in any way.

Are there any benefits to me?

You may receive direct benefits by participating in the Study. The results of the research could also benefit others in the future. This study is examining alternative ways for accessing medication for a common health issue. It will help inform if this is a safe and effective strategy.

Are there any risks to me?

The risk to you is low. We are collecting information on your medication and health service usage. Once the data is obtained it will only be analysed with identifiers removed.

Sources of funding

The Study is funded by New South Wales Ministry of Health.

What if I have more questions?

If you have any questions about the Study, or would like any additional information before deciding to participate, please contact the study team on 02 8052 4300 or email uti@georgeinstitute.org

What can I do if I have a complaint?

The University of Newcastle Human Research Ethics Committee (HREC No.xxx) has approved this study based on strict ethical standards and security of participant confidentiality and privacy. If you have any complaints or concerns about the manner in which this research is being conducted, please contact Human-Ethics@newcastle.edu.au or contact the Office of the Australian Information Commissioner at enquiries@oaic.gov.au or tel: 1300 363 992

If you have a privacy complaint in relation to the use of your Services Australia information, you should contact the Office of the Australian Information Commissioner. You will be able to lodge a complaint with them.

www.oaic.gov.au
1300 363 992 enquiries@oaic.gov.au
GPO Box 5218, Sydney NSW 2001

Your personal information Services Australia hold is protected by the Privacy Act 1988 and cannot be given to a third party without your consent or where otherwise permitted by law. For more information about privacy, go to servicesaustralia.gov.au/privacy

Thank you for your time.

Australian Government
Services Australia

Study Participant ID:

Participant Consent Form for the release of Services Australia information

(Participants over 14 years of age only)

Consent to release of my Medicare Benefits Schedule (MBS) and/or Pharmaceutical Benefits Scheme (PBS) information by Services Australia to The George Institute for Global Health for the purpose of the Management of Urinary Tract Infections by Community Pharmacists Study)

Important Information

Complete this form to request the release of your MBS claims information and/or PBS claims information to the study. The signatory must initial any changes to this form. Incomplete forms may result in the study not being provided with your information.

Rights and Privacy (tick all relevant boxes to indicate fully informed consent)

I understand:

- ☐ my MBS and/or PBS will be disclosed by Services Australia for the purpose of the study.
- ☐ the results of this research may be published in articles or journals.
- ☐ my name will never be disclosed by Services Australia, used in the study or published.
- ☐ my participation in the study is completely voluntary.
- ☐ I can withdraw my consent to release my Services Australia information to the study at any time (refer to the participant information sheet and withdrawal of consent form), and I do not have to provide a reason.
- ☐ the information provided to me about the study, and I have been given the opportunity to ask questions, and any questions I have asked have been answered to my satisfaction.

Consent:

☐ I consent to the disclosure by Services Australia of my MBS and/or PBS information to researchers for the purposes of the study

Participant Details

☐ Mr ☐ Mrs ☐ Ms ☐ Miss ☐ Other ____________

First name ____________ Family name ____________

other given name(s) ____________

Date of birth:

DD / MM / YYYY

Medicare card number (first 9 digits only):

e.g. 123453789

Primary address:

Postal address:

Authorisation:

I authorise Services Australia to provide my:

- ☐ MBS claims history OR
- ☐ PBS claims history OR
- ☐ MBS & PBS claims histories

For the period * ___ /____ /_____ to: ___ /___ /_____ to the study.
Date range is to be completed prior to or at the time of signing the consent form.

*Note: As Services Australia can only extract 4.5 years of data (prior to the extraction), the consent period above may result in multiple extractions.

☐ If in the event that I pass away during the study period, I consent to Services Australia to continue to provide my claims information to the study.

Declaration:

I declare that the information on this form is true and correct.

Sign and date:

DD/MM/YYYY

If signed by a Legal Guardian/POA other than the participant please print name, sign & date below:

First name	Second name
Signature	DD/MM/YYYY

- ☐ Legal Guardian (where the participant is over the age of 14 years old)
- ☐ Power of Attorney
- ☐ Guardianship order/Administration order

Please attach supporting evidence (Power of Attorney document **(medical or enduring)** or legal guardianship)

Once a young person has turned 14 years old they must consent to their own information being released.

Consent forms will not be processed without the relevant supporting evidence.

Power of attorney – A power of attorney is a document that appoints a person to act on behalf of another person who grants that power. In particular, an enduring power of attorney allows the appointed person to act on behalf of another person even when that person has become mentally incapacitated. The powers under a power of attorney may be unlimited or limited to specific acts.

Guardianship/Administration order – A Guardianship/ Administration order is an order made by a Guardianship Board/Tribunal that appoints a guardian to make decisions for another person. A Guardianship order may be expressed broadly or limited to particular aspects of the care of another person.

A sample of the information that may be included in your MBS claims history:

Date of service	Date of Processing	Item number	Item description	Provider charge	Schedule Fee	Benefit paid	Patient out of pocket
20/04/09	03/05/09	00023	Level B consultation	$38.30	$34.30	$34.30	$4.00
22/06/09	23/06/09	11700	ECG	$29.50	$29.50	$29.50	

Date of referral	Hospital Indicator	Item category
	N	1
20/04/09	N	2

A sample of the information that may be included in your PBS claims history:

Date of supply	Date of prescribing	PBS item code	Item description	Patient category	Patient contribution (this includes under co-payment amounts**)
06/03/09	01/03/09	03133X	Oxazepam Tablet 30 mg	Concessional Ordinary	$5.30
04/07/09	28/05/09	03161J	Diazepam Tablet 2 mg	General Ordinary	$30.85

Pharmacy postcode	ATC Code	ATC Name
2560	N05 B A 04	Oxazepam
2530	N05 B A 01	Diazepam

** Under co-payments can now be provided for data after 1 July 2012

Privacy and your personal information

The privacy and security of your personal information is important to us, and is protected by law. We need to collect this information so we can process your applications and payments, and provide services to you. We only share your information with other parties where you have agreed, or where the law allows or requires it. For more information, go to **servicesaustralia.gov.au/privacy**

Dec 2021

Australian Government
Services Australia

Services Australia Participant Withdrawal of Consent Form

The Management of UTIs by Community Pharmacists Study

I wish to WITHDRAW my consent to release my Services Australia information to the study effective from the date below. I request that the study handles the information they have collected about me in the following way (choose one option):

☐ DESTROY all information collected about me to date so it can no longer be used for research

☐ RETAIN all information collected about me to date so it can continue to be used for research

I understand that:

1. no further information about me will be provided to the study from the withdrawal date;
2. information about me that has already been analysed and/or included in a publication by the study, may not be able to be destroyed; and
3. choosing to withdraw my information from the study will not affect my access to Health Services or Government benefits.

Print first/second name, signature & date

First name
Second name
Signature
DD/MM/YYYY

This form should be forwarded by email to: uti@georgeinstitute.org. Alternatively, forms can be posted to:

Valerie Looi, Project Manager, The George Institute for Global Health,

Level 5, 1 King St Newtown, NSW 2042
