# Supplementary material for: Pathway to Access UTI Management (PATH-UTI): evaluating pharmacist prescribing and management practices for uncomplicated urinary tract infections in community pharmacy in New South Wales and the Australian Capital Territory, Australia
Source: Int J Clin Pharm. 2026 Sep 23;48(6):2490–503. doi: 10.1007/s11096-026-02226-9 (PMC13731454; doi:10.1007/s11096-026-02226-9)
Supplement: Supplementary file 3 [file 11096_2026_2226_MOESM3_ESM.pdf]

PATH-UTI
PATHWAY TO ACCESS: UTI MANAGEMENT

# Clinical Management Protocol (Main Evaluation Trial)

## Management of Urinary Tract Infections by Community Pharmacists

Sex of patient

- Male or individuals who have undergone gender reassignment surgery → Yes → Exclusion from trial: Usual care is provided by pharmacist and/or referral to a medical professional based on presenting symptoms

Female

↓ Yes

Aged 18-65 years → No → Exclusion from trial

↓ Yes

Patient consents (patient must physically be present in the pharmacy) → No → Exclusion from trial

↓ Enrol into trial

Systemic symptoms suggesting pyelonephritis
(e.g., fever >38°C, chills, nausea, malaise, flank pain)
Suspected sepsis (e.g., patient with fever, difficulty breathing, fast heart rate, low blood pressure and mental confusion)

→ Yes → **Immediate referral to Emergency Department**

↓ No

- Pregnancy or 4-6 weeks post-partum
- Recurrent UTI (2 or more within 6 months or 3 or more within 12 months)
- Symptoms of UTI persisting 48-72 hours after starting appropriate antibiotic treatment
- Symptoms of UTI reoccur within 2 weeks after finishing appropriate antibiotic treatment
- Visible haematuria (blood in urine)
- Pyuria (pus in urine)
- Renal disease, impaired renal function and history of renal stones
- Catheterisation
- Previous episodes of pyelonephritis
- Immunocompromised or taking immunosuppressant medicines (e.g., azathioprine, cyclosporin, cyclophosphamide, methotrexate [including low dose])
- History of blood disorder/porphyria
- Spinal cord injury
- Diabetes
- Recent insertion of IUD within the last 3 months

→ Yes → **Immediate referral to GP**

Only conservative management with non-prescription medicines (if appropriate) and self-care advice may be provided by pharmacist prior to review by a medical practitioner

↓ No

- Symptoms or medical history suggest a cause other than acute UTI – (e.g., vaginal thrush)

→ Yes → **Treatment and/or referral to GP based on presenting symptoms**

↓ No

Presenting with any other symptoms or complications

- Multidrug-resistance bacteria-risk factors including:
  - Recent (within the previous 3 months) or frequent treatment with antibiotics
  - Recent (within the previous 3 months), frequent or long-term resident of a healthcare facility, history of being hospitalised in the last 4 weeks
  - Overseas travel within the previous 3 months, or overseas travel within previous 6 months to regions with high prevalence of antimicrobial resistance (e.g., South Asia, South-East Asia) particularly if the patient has received medical care or treatment with antibiotics in that region
  - Infection with multidrug-resistant bacteria within the previous 3 months
- Risk factors for sexually transmissible infection
  - Age < 29 years or previous STI, or sexual contact without a condom/dental dam outside a mutually monogamous relationship, a new sexual partner in last 60 days, a sex partner recently treated for an STI, sexual contact with a sex worker
- Currently taking medicines that increase the risk of UTI, e.g., SGLT2 inhibitors
- IUD in situ

→ Yes → **Treat using conservative measures or antibiotic treatment, if clinically appropriate. Refer the patient to a GP for review after provision of treatment by the pharmacist**

↓ No - continued

* Protocol developed and agreed as part of co-design with Project Partners

THE UNIVERSITY OF NEWCASTLE AUSTRALIA

PATH-UTI
PATHWAY TO ACCESS: UTI MANAGEMENT

# Clinical Management Protocol (Main Evaluation Trial)

## Management of Urinary Tract Infections by Community Pharmacists

No - continued

**Signs of uncomplicated UTI (at least 2 of: dysuria, urinary frequency, urinary urgency, suprapubic pain or discomfort)**

→ No → **Only one primary symptom of uncomplicated UTI (i.e., one symptom of dysuria, frequency, urgency, suprapubic pain or discomfort – one symptom is not sufficient to indicate presence of a UTI)** → Yes → **Conservative management by pharmacist only**

→ Yes ↓

**Provisional diagnosis for uncomplicated UTI**

↓

**Proceed to appropriate treatment**

↓ Yes

Check allergies and any contraindications before providing treatment → Yes → **Treat and consider antibiotic choice if appropriate and/or referral to GP**

↓

**First line: Provide conservative management with non-prescription medicines as first line treatment and ensure evidence of use**

Analgesia: Paracetamol or non-steroidal anti-inflammatory drugs (e.g., ibuprofen, naproxen) can reduce the pain and discomfort of UTI

- Paracetamol: 1g orally, every 4- 6 hours as required. Maximum dose of 4g in 24 hours, or
- Ibuprofen: 200-400mg orally, every 6-8 hours as required. Maximum daily dose of 2.4g in 24 hours

Urinary alkalinising agents – noting safety and efficacy of these agents for the symptomatic treatment of UTI have not been established

↓

**Provide non-pharmacological and self-care advice**

Consumer Medicines Information and/or a Self-Care Fact Card

Identify and educate patients who are prone to developing UTIs and provide advice on prophylaxis (i.e., increase fluid intake to 2-3L daily)

↓

**Provide, if required, appropriate antibiotic treatment to the patient**

- First line - **Trimethoprim 300mg** orally, daily at night for 3 nights (Supply 3 tablets)
- Second line - **Nitrofurantoin 100mg** orally, every 6 hours for 5 days (Supply 20 tablets)
- Third line - **Cefalexin 500mg** orally, every 12 hours for 5 days (Supply 10 tablets)

Consider drug allergies and drug interactions when selecting treatments (e.g., Nitrofurantoin interacts with urinary alkalinising agent)

→ Yes → **Dispense any medications (if supplied) via pharmacy dispensing software and label according to the legislative requirements outlined in the Poisons and Therapeutic Goods Regulation 2008**

↓

**Provide follow up advice and expectations around duration of symptoms**

- Symptoms should respond to appropriate antibiotic treatment within 48 hours
- Provide patient with a urine specimen jar. Advise patient to take a urine sample before commencing first dose of antibiotic treatment, refrigerate and take sample with them to see a GP if their symptoms worsen or no improvement in 48 hours. If symptoms persist 48–72 hours after starting appropriate antibiotic treatment, symptoms reoccur within 2 weeks after finishing antibiotic treatment or symptoms develop that are not symptoms of an acute UTI, patient should be advised to see a GP

→ **Document the consultation in MedAdvisor and share a record of the consult with the patient's usual treating medical practitioner or medical practice**

**Note:** If no GP or hospital service is available for the patient, referral should be made to **HealthDirect** via 1800 022 222
