# Supplementary material for: Pathway to Access UTI Management (PATH-UTI): evaluating pharmacist prescribing and management practices for uncomplicated urinary tract infections in community pharmacy in New South Wales and the Australian Capital Territory, Australia
Source: Int J Clin Pharm. 2026 Sep 23;48(6):2490–503. doi: 10.1007/s11096-026-02226-9 (PMC13731454; doi:10.1007/s11096-026-02226-9)
Supplement: Supplementary file 4 [file 11096_2026_2226_MOESM4_ESM.pdf]

Patient follow up data collection template

Dear <insert name>, you recently consulted your pharmacist, at [pharmacy name] on [date] and took part in a trial to look at management of Urinary Tract Infections by community pharmacists.

We are keen for feedback on this service. There are just a few questions, and it will take 5 minutes to complete. Please click on this link <insert link> to fill out the survey.

Researcher ID

____

Pharmacy ID

______________________________ **(This ID should match with the ID provided by MedAdvisor)**

Patient ID

______________________________ **(This ID should match with the ID provided by MedAdvisor)**

Date of pharmacy consultation

____

Date/time of patient follow up interview

____

1. How would you best describe your urinary symptoms today?

☐ My symptoms have completely resolved

If yes:

How many days did it take for your symptoms to completely resolve after seeing your pharmacist?

______________________________ **days**

☐ My symptoms have improved, but not completely resolved
☐ My symptoms have not improved or worsened

(Researcher to advise to urgently seek advice from a health care professional if symptoms have not improved or have worsened)

2. Were you prescribed an antibiotic for your symptoms?

☐ Yes ☐ No

If yes:

Did you take your antibiotic doses at the specified times for the recommended duration of treatment?

☐ Yes ☐ No

For how many days did you take your antibiotic? ______ days

Did you have any difficulties in taking your antibiotics?

☐ Yes ☐ No

If yes, what difficulties did you experience? _____________________

3. **Did you experience any side effects or other complications, including from medications?**

☐ Yes ☐ No ☐ Don't know

If yes:

Which side effects or other complications did you experience? (select all that apply)

- ☐ Nausea
- ☐ Vomiting
- ☐ Diarrhoea/ Loose stools
- ☐ Constipation
- ☐ Skin rash
- ☐ Difficulty breathing
- ☐ Fatigue
- ☐ Other

If other, please specify:

____

Have your complications resolved?

☐ Yes ☐ No

(If no, then study team will follow up with reporting procedure as per protocol)

4. **Did you use any other products/medicines for your urinary symptoms?**

☐ Yes ☐ No

If yes:

Select all that apply:

- ☐ Urinary alkalinizer
- ☐ Cranberry juice or capsules
- ☐ Vitamin C
- ☐ Oral pain relief (e.g., paracetamol or ibuprofen)
- ☐ Other

If other, please specify:

____

5. **Did you see another health care professional for the same symptoms in the last 7 days?**

☐ Yes ☐ No

If yes:

Which of the following health services did you attend? (select all that apply)

☐ The same pharmacy that I got my original treatment from
☐ A different pharmacy
☐ A general practitioner
☐ The hospital emergency department*
☐ Urgent care clinic
☐ Health information/nurse phone line like Health Direct
☐ Other

If other, please specify:

____

Did you receive any medications from any of these health care professionals for your symptoms?

☐ Yes ☐ No

If yes, please specify which ones: _____________________

Were you admitted to hospital for any reason in the last 7 days?

☐ Yes ☐ No

6. **Approximately how much did the whole treatment at the pharmacy cost you?**

[provide amount in $ including any consultation fee, prescribed medications and other medications recommended to you for this condition]

7. **Would cost be a barrier to your using this service again?**

☐ Yes ☐ No ☐ Maybe

8. **How much do you agree or disagree with the following statements:**

   1. Overall, I am satisfied by the consultation provided by the pharmacist for my urinary symptoms

	1	2	3	4	5	6	7	
Strongly disagree	☐	☐	☐	☐	☐	☐	☐	Strongly agree

2. The staff at this pharmacy have the knowledge to answer my questions about my urinary symptoms

	1	2	3	4	5	6	7	
Strongly disagree	☐	☐	☐	☐	☐	☐	☐	Strongly agree

3. I am confident that this pharmacy dispenses medication correctly

	1	2	3	4	5	6	7	
Strongly disagree	☐	☐	☐	☐	☐	☐	☐	Strongly agree

4. I am satisfied with the amount of privacy I received at the pharmacy

	1	2	3	4	5	6	7	
Strongly disagree	☐	☐	☐	☐	☐	☐	☐	Strongly agree

5. It was more convenient for me to obtain care for my urinary symptoms from a pharmacist compared with another care provider

	1	2	3	4	5	6	7	
Strongly disagree	☐	☐	☐	☐	☐	☐	☐	Strongly agree

6. I think the care provided by the prescribing pharmacist for my urinary symptoms was as good as my usual care

	1	2	3	4	5	6	7	
Strongly disagree	☐	☐	☐	☐	☐	☐	☐	Strongly agree

7. The pharmacists allowed me an opportunity to be involved in making decisions about my care

	1	2	3	4	5	6	7	
Strongly disagree	☐	☐	☐	☐	☐	☐	☐	Strongly agree

Some additional information about you:

What is your highest educational attainment?

☐ Postgraduate Degree
☐ Graduate Diploma/Graduate Certificate
☐ Bachelor Degree
☐ Advanced Diploma/Diploma
☐ Year 12 or equivalent
☐ Year 10 or equivalent
☐ Year 9 or below
☐ Never attended school
☐ Prefer not to say

How would you describe your employment status?

☐ Employed, working full-time (if usually work 35 hours or more a week (in all jobs))
☐ Employed, working part-time (if usually work one hour to less than 35 hours a week (in all jobs))
☐ Unemployed, looking for full-time OR part-time work (actively seeking full-time work in last 4 weeks)
☐ Not in the labour force
☐ Other
☐ Prefer not to say

Are you of Aboriginal and/or Torres Strait Islander descent?

☐ Yes, Aboriginal
☐ Yes, Torres Strait Islander
☐ Yes, Aboriginal and Torres Strait Islander
☐ No
☐ Prefer not to say

<u>For researchers only:</u>
Estimated time taken to complete patient follow up interview

______________________________ mins

Thank you for your time completing this survey
