# Supplementary material for: Pathway to Access UTI Management (PATH-UTI): evaluating pharmacist prescribing and management practices for uncomplicated urinary tract infections in community pharmacy in New South Wales and the Australian Capital Territory, Australia
Source: Int J Clin Pharm. 2026 Sep 23;48(6):2490–503. doi: 10.1007/s11096-026-02226-9 (PMC13731454; doi:10.1007/s11096-026-02226-9)
Supplement: Supplementary file 5 [file 11096_2026_2226_MOESM5_ESM.pdf]

Table 1. Incidence of adverse events for trimethoprim in 9 databases and the PATH-UTI trial* (supplementary material for detailed ADRs per antibiotic and summary of most common/prevalent ADRs).

**ICD-10 category**	**Adverse event**	**Vigibase % (WHO, Uppsala Monitoring Centre From 12,691 reports)**	**TGA DAEN % (From 1,306 reports)**	**Micromedex % Range**	**CIMA (EMA) % Range (unless lower 0.01% or less)**	**Drugs.com % Range**	**UpToDate % or % Range**	**Mayo Clinic % Range**	**NHS % Range**	**PI/CMI % or % Range**	**PATH-UTI Trial Reported Number (n)**	**PATH-UTI Trial % of adverse events at follow up (13,260 total surveys)**	**Risk category**
**Dermatological**	Night sweats	0.05	0.16								2	0.015	
	Skin rash	20	51	1-10	1-10	1-10	7 (1-10)	0.1-1	1-10	8 (1-10)	74	0.56	
	Sunburn	0.5	0.5								1	0.007	
**Cardiovascular**	Tachycardia	0.41	0.84								2	0.015	
**Gastrointestinal**	Abdominal pain	3	2		1-10		0.1-1	0.1-1			31	0.23	
	Bloating	0.11	0.1								11	0.08	
	Constipation	0.5	0.4			0.01-0.1			0.01-0.1	0.01-0.1	65	0.49	

	Diarrhoea	2	2			1-10	4 (1-10)	0.1-1	1-10	1-10	109	0.82	
	Dry mouth	0.31	0.38								2	0.015	
	Dysgeusia	0.2	0.2		1-10						5	0.038	
	Loss of appetite	0.78	1.0					0.1-1		0.01-0.1	2	0.015	
	Mouth ulcers	1.1	1.5								2	0.015	
	Nausea	6	7		1-10	1-10		0.1-1	1-10		282	2.1	
	Oral candidiasis	0.04									5	0.038	
	Oral pain	0.28	0.08								2	0.015	
	Vomiting	5	6		1-10		2 (1-10)	0.1-1	1-10		37	0.27	
**Genitourinary**	Dysuria	0.13	0.16								2	0.015	
	Haematuria	0.2	0.08			0.01-0.1		0.01-0.1	0.01-0.1	0.01-0.1	2	0.015	
	Lack of efficacy	0.61	0.94								30	0.22	
	Thrush	0.2	0.2			1-10			1-10	1-10	114	0.86	
**Immunological**	Allergic reaction	3.7	1		0.01	0.01-0.1		0.01-0.1	0.01-0.1	0.01-0.1	10	0.075	

	Anaphylaxis	2.5	3.4		0.01	0.01-0.1		0.01-0.1	0.01-0.1	0.01-0.1	1	0.007	
**Neurological**	Fatigue	3.5	4.2			0.01-0.1			0.01-0.1		117	0.88	
	Headache	4	3			1-10		0.01-0.1	1-10	1-10	44	0.33	
	Somnolence	0.5	0.3			0.01-0.1			0.01-0.1		3	0.023	
**Ophthalmological**	Blurred vision	0.23	0.47								2	0.015	
**Psychiatric**	Claustrophobia	0.02	0.07								1	0.007	
	Depression-like symptoms	0.2	0.2								5	0.038	
	Hallucinations	0.63	0.53							0.01-0.1	1	0.007	
	Parasomnia	0.5	0.2			0.01-0.1			0.01-0.1	0.01-0.1	6	0.045	
**Respiratory**	Difficulty breathing	2	0.3			0.01-0.1		0.01-0.1	0.01-0.1	0.01-0.1	18	0.14	
	Flu-like symptoms	1.5	2			0.01-0.1		0.01-0.1	0.01-0.1	0.01-0.1	3	0.023	

**Other**	Body pain	1.5	2.5								11	0.08	
	Epistaxis	0.1	0.07			0.01-0.1			0.01-0.1		1	0.007	
	Fever	5	5								3	0.023	
	Flushing	0.3	1.3								1	0.007	

***Note: The databases report incidence of adverse events to a different number of decimal places.**

Table 2. Incidence of adverse events for nitrofurantoin in 9 databases and the PATH-UTI trial*

ICD-10 category	Adverse event	Vigibase % (WHO, Uppsala Monitoring Centre From 12,691 reports)	TGA DAEN % (From 1,306 reports)	Micromedex % Range	CIMA (EMA) % Range (unless lower 0.01% or less)	Drugs.com % Range	UpToDate % or % Range	Mayo Clinic % Range	NHS % Range	PI/CMI % or % Range	PATH-UTI Trial Reported Number (n)	PATH-UTI Trial % of adverse events at follow up (256 total surveys)	Risk category
**Dermatological**	Skin rash	27	23	1-10	0.1-1		0.1-1	1-10			2	0.8	
**Gastrointestinal**	Abdominal pain	6	5	1-10	1-10	0.1-1	0.1-1	0.01-0.1		0.1-1	0	0	
	Bloating	0.7	0.25			1-10	2 (1-10)	1-10			1	0.4	
	Constipation	0.5	0.25			0.1-1	0.1-1				6	2.3	
	Diarrhoea	4	4		1-10	0.1-1	0.1-1	1-10	1-10	0.1-1	8	3.1	
	Loss Appetite	2.1	3.7								2	0.8	
	Nausea	12	13	1-10	1-10	1-10	8 (1-10)	1-10	1-10	1-10	22	8.6	
	Vomiting	8	10	1-10	1-10	1-10	0.1-1	0.01-0.1	1-10	1-10	1	0.4	

**Genitourinary**	Haematuria	1	0.5					0.1-1	0.01-0.1		2	0.8	
	Lack of efficacy	1.7	0.4								1	0.4	
	Thrush	0.3	0.4								2	0.8	
**Immunological**	Anaphylaxis	1	0.7					0.01-0.1	0.01-0.1		1	0.4	
	Allergic reaction	5	1.4					0.01-0.1	0.01-0.1		0	0	
**Neurological**	Drowsiness	1.5	1			0.1-1	0.1-1	0.1-1	1-10		0	0	
	Fatigue	8.7	9		1-10	0.1-1	0.1-1	0.1-1			8	3.1	
	Headache	8	7			1-10	6 (1-10)	0.1-1	1-10		2	0.8	
**Respiratory**	Difficulty breathing	9	0.5		0.01		0.1-1	1-10	0.01-0.1		2	0.8	
	Flu-like symptoms	5	6.5		0.01		0.1-1	1-10	0.01-0.1		3	1.2	

Note: The databases report incidence of adverse events to a different number of decimal places.

Table 3. Incidence of adverse events for cefalexin in 9 databases and the PATH-UTI trial*

ICD-10 category	Adverse event	Vigibase % (WHO, Uppsala Monitoring Centre From 12,691 reports)	TGA DAEN % (From 1,306 reports)	Micromedex % Range	CIMA (EMA) % Range (unless lower 0.01% or less)	Drugs.com % Range	UpToDate % or % Range	Mayo Clinic % Range	NHS % Range	PI/CMI % or % Range	PATH-UTI Trial Reported Number (n)	PATH-UTI Trial % of adverse events at follow up (368 total surveys)	Risk category
**Dermatological**	Skin rash	60	31	0.1-1	0.1-1	0.01-0.1		0.1-1	0.1-1	0.1-1	4	1.1	
**Gastrointestinal**	Abdominal pain	9	4		0.01-0.1	0.1-1		1-10	0.1-1	1-10	0	0	
	Bloating	0.16	0.1								1	0.3	
	Constipation	0.43	0.2								2	0.5	
	Diarrhoea	6.2	6		1-10	1-10		1-10	1-10	1-10	5	1.4	
	Nausea	5	5.5	1-10	1-10	0.1-1		1-10	0.1-1	0.01-0.1	4	1.1	
	Vomiting	4.4	5		0.01-0.1	0.1-1			0.1-1	0.01-0.1	0	0	
**Genitourinary**	Haematuria	0.2	0.2						0.1-1		0	0	

	Lack of efficacy	0.9	0.6		0.01-0.1						0	0	
	Thrush (fungal superinfection)	0.48	0.5		0.01-0.1						7	1.9	
**Immunological**	Allergic reaction	8.6	2		0.01-0.1			0.1-1		0.01-0.1	0	0	
	Anaphylaxis	3.5	7		0.01-0.1			0.1-1		0.01-0.1	0	0	
**Neurological**	Fatigue	3.8	2		0.01-0.1	0.01-0.1			0.1-1		2	0.5	
	Headache	2.2	1			0.01-0.1			0.1-1		0	0	
**Respiratory**	Difficulty breathing	3.7	0.04			0.01-0.1					1	0.3	
	Flu-like symptoms	1.2	0.7			0.01-0.1			0.1-1		0	0	

Note: The databases report incidence of adverse events to a different number of decimal places.
